# A Machine Learning Approach to Prioritizing Functionally Active *F-box* Members in *Arabidopsis thaliana*

**DOI:** 10.3389/fpls.2021.639253

**Published:** 2021-05-28

**Authors:** Yang Li, Madhura M. Yapa, Zhihua Hua

**Affiliations:** ^1^Department of Environmental and Plant Biology, Ohio University, Athens, OH, United States; ^2^Interdisciplinary Program in Molecular and Cellular Biology, Ohio University, Athens, OH, United States

**Keywords:** Arabidopsis, *F-box*, UPS, activity, machine leaning, artificial neural network, expression, evolution

## Abstract

Protein degradation through the Ubiquitin (Ub)-26S Proteasome System (UPS) is a major gene expression regulatory pathway in plants. In this pathway, the 76-amino acid Ub proteins are covalently linked onto a large array of UPS substrates with the help of three enzymes (E1 activating, E2 conjugating, and E3 ligating enzymes) and direct them for turnover in the 26S proteasome complex. The S-phase Kinase-associated Protein 1 (Skp1), CUL1, F-box (FBX) protein (SCF) complexes have been identified as the largest E3 ligase group in plants due to the dramatic number expansion of the *FBX* genes in plant genomes. Since it is the FBX proteins that recognize and determine the specificity of SCF substrates, much effort has been done to characterize their genomic, physiological, and biochemical roles in the past two decades of functional genomic studies. However, the sheer size and high sequence diversity of the *FBX* gene family demands new approaches to uncover unknown functions. In this work, we first identified 82 known *FBX* members that have been functionally characterized up to date in *Arabidopsis thaliana*. Through comparing the genomic structure, evolutionary selection, expression patterns, domain compositions, and functional activities between known and unknown *FBX* gene members, we developed a neural network machine learning approach to predict whether an unknown *FBX* member is likely functionally active in Arabidopsis, thereby facilitating its future functional characterization.

## Introduction

Since the first group of land plants emerged on the earth, various harsh conditions such as drought, severe temperatures, soil salinity, pathogen and insect infections, and herbivore attacks have become inevitable living conditions. The climate changes and humanmade environmental damages have been adding more challenges to plant growth and survival. To cope with an ever-changing living environment, the sessile lifestyle requires plants to carry out rapid adjustment of internal metabolic pathways to percept, transduce, and respond to numerous internal and external cues. Since the first genome of *Arabidopsis thaliana* (Arabidopsis hereafter) was obtained in 2000 (Arabidopsis Genome Initiative, 2000), numerous genome sequencing projects have exclusively demonstrated one robust metabolic regulatory machinery that is composed of a large group of members in plant genomes. This machinery is called the Ubiquitin (Ub)-26S Proteasome System (UPS).

The UPS is designed to regulate protein functions post-translationally. The entire system can be spatially and temporarily divided into two tandem biochemical pathways, ubiquitylation and degradation. Ubiquitylation of protein substrates usually takes place by a three-step cascade biochemical reaction that involves one common Ub-activating (E1) enzyme, few Ub-conjugating (E2) enzymes, and a large group of Ub ligases (E3) (Hershko and Ciechanover, 1998; Vierstra, 2009; Hua and Vierstra, 2011; Finley et al., 2012; Marshall and Vierstra, 2019). In general, it is the E3 ligases that determine whether a ubiquitylation substrate is routed into the UPS regulation. Protein ubiquitylation results in the changes of activity and/or intracellular locations of a substrate, but in many cases, leads a substrate to turn over. If a ubiquitylation substrate is tagged by poly-Ub chains, in which the Ub moieties are connected through their 11th or 48th lysine (K11/K48) residues, it will be recognized by the 26S proteasome for degradation (Kim et al., 2013; Yau and Rape, 2016; Marshall and Vierstra, 2019). Although emerging data suggested that the 26S proteasome and Ub-conjugating enzymes could change the fate of a ubiquitylation substrate (Kataria et al., 2014; Marshall and Vierstra, 2019), the biochemical functions of E3 ligases have been well appreciated for their specific roles in recruiting protein substrates into the UPS regulatory pathway (Vierstra, 2009; Hua and Yu, 2019). It has been estimated that an equally large group of E3 ligases and ubiquitylation substrates are encoded in plant genomes.

However, due to the challenge in identifying many short-lived and low abundant ubiquitylation substrates, the essential regulatory roles of the plant UPS were initially recognized by the large group of E3 ligases encoded in plant genomes. Because many E3 ligases are composed of protein families that share common protein-protein interaction domains, identifying E3 ligases is relatively easier than characterizing ubiquitylation substrates. For example, right after the first draft of Arabidopsis genome was sequenced in 2000 (Arabidopsis Genome Initiative, 2000), 695 *F-box* (*FBX*) genes were identified in this plant (Gagne et al., 2002). Since then, the completion of more plant genome sequencing projects has revealed that the UPS, particularly the E3 ligase group, has dramatically expanded in land plants compared to any other eukaryotic organisms. It has been suggested that the large expansion of the UPS is important for plants to cope with environmental changes (Grau-Bove et al., 2015).

The *FBX* genes encode a protein that contains at least two distinct protein-protein interaction modules, an N-terminal FBX domain (FBXD) and a C-terminal substrate recognition module. Through interacting with the S-phase Kinase-associated Protein 1 (Skp1) via the FBXD, all the functionally active FBX proteins assemble a Skp1-CUL1-FBX (SCF) multi-subunit E3 ligase complex. In this complex, CUL1 plays a scaffold role to dock the heterodimeric Skp1-FBX proteins at its N-terminus and a Really Interesting New Gene Box 1 (RBX1) at its C-terminus. During the ubiquitylation process, a Ub-conjugating enzyme associates with RBX1 in the complex to bring an activated Ub in close proximity to the SCF substrate that is recruited through interacting with the C-terminal substrate recognition module of the FBX protein (Zheng et al., 2002). Such a structural design results in the formation of an isopeptide bond between the carboxyl group of the C-terminal glycine residue of the Ub and the ε-amino group of a lysine residue on the substrate. Due to the presence of seven lysine residues on Ub, multiple Ubs can be conjugated sequentially with a preceding Ub moiety to form a poly-Ub chain. Although it is yet unknown whether SCF E3 ligases catalyze a specific type of ubiquitylation reaction, various structural topologies of poly-Ub chains can occur if the Ub moieties are conjugated through different lysine residues (Welchman et al., 2005; Yau and Rape, 2016). Hence, the resulting ubiquitylated substrates can have different fates either changing functions or being recognized by the 26S proteasome for degradation. Through the past two decades of functional genomic studies in Arabidopsis, a handful of *FBX* genes have been either phenotypically or biochemically characterized. To date, all the known SCF substrates in Arabidopsis are ultimately turned over by the 26S proteasome, suggesting that the plant-type SCF complexes primarily mediate the polyubiquitylation of their substrates via K48 or K11 on the Ub moieties. Through these studies, SCF complex-mediated ubiquitylation has been demonstrated to regulate a wide range of developmental processes, from early seed germination (Ariizumi et al., 2011; Majee et al., 2018), photomorphogenesis (Quint et al., 2005; Moon et al., 2007; Gilkerson et al., 2009; Yapa et al., 2020), circadian rhythms (see review by Johansson and Staiger, 2015), cell cycle (del Pozo et al., 2006; Kim et al., 2008; Noir et al., 2015) to late floral organ establishment (Zhao et al., 1999; Durfee et al., 2003; Yapa et al., 2020), self-incompatibility (Hua et al., 2008; Li and Chetelat, 2015; Sun et al., 2018), and embryogenesis/seed development (Liu et al., 2004; Yapa et al., 2020). In addition, SCF complexes are also known to play important roles in stress responses (Cheng et al., 2011; Hedtmann et al., 2017; Doroodian and Hua, 2021) and hormone signaling (see reviews by Santner and Estelle, 2010; Hua and Vierstra, 2011). Recently, we discovered a new role of SCF^CFK1^ complex in epigenetic regulation by controlling the stability of *de novo* DNA methyltransferase (Chen et al., 2020).

Given the large size of the Arabidopsis *FBX* gene superfamily, the number of characterized members is significantly lower than those in many other angiosperm core gene families (Li et al., 2016). To tackle this puzzle, several research groups have been studying the genomic and evolutionary features of this unique gene family. Through phylogenetic studies, Gagne et al. (2002) first discovered that the Arabidopsis *FBX* genes can be phylogenetically separated into 20 distinct groups with various sizes. Given the diverse FBXD sequences, Gagne et al. (2002) hypothesized that different groups of FBX proteins may preferentially bind to different Arabidopsis Skp1 like (ASK) proteins. However, direct biochemical and *in vivo* functional evidence that may support this hypothesis is yet lacking. The increasing number of sequenced plant genomes make it possible to carry out comparative genomic studies of the plant *FBX* gene family. Through size comparison among Arabidopsis, rice, and Populus, Yang et al. (2008) concluded that herbaceous annual plants encoded more *FBX* genes than woody perennial plants, arguing that the fewer *FBX* genes in Populus are integral to its biological processes. However, through further careful comparison across 18 plant genomes, ranging from the green alga *Chlamydomonas reinhardtii* to numerous monocots and eudicots, we disagreed with this conclusion. Instead, the results of our studies are in favor of a genomic drift evolutionary theory, by which the plant *FBX* gene family could expand in a process that is not related to the complexity of a plant species. For example, *Zea may*s and *Sorghum bicolor* are two closely related monocotyle species that split 12 million years ago (mya). However, *S. bicolor* encodes a greater than twofold of the number of *FBX* genes predicted in *Z. mays* (Hua et al., 2011). Subsequent studies in 443 Arabidopsis populations allowed us to discover that the expression of a large group of Arabidopsis *FBX* genes is epigenetically suppressed and their coding sequences are undergoing a rapid process of pseudogenization (Hua et al., 2013). Xu et al. (2009) discovered that unusually frequent shifts of exon-intron boundaries and/or frameshift mutations resulted in the size variance of the *FBX* gene families in Arabidopsis, poplar, and rice. Without further expression and functional studies, the adaptive role of *FBX* sequence divergence in plants is hypothetic. Recently, through genomic comparison of a large number of plant genomes (in total, 111 species), we discovered four clusters of plant *FBX* genes that experienced different retention rates, functional constraints, and phylogenetic distributions. This discovery allowed us to develop purifying and dosage balancing selection models for the evolution of plant *FBX* genes. Because lineage/species-specific *FBX* genes are detrimental due to the activation of unwanted degradation of numerous substrates, these members are kept in low frequencies in plant genomes, a phenomenon similar to the frequency suppression of detrimental alleles by purifying selection in populations. Therefore, in analogy to the purifying selection on detrimental alleles, we adapted the term *purifying selection* to explain the frequency suppression of *FBX* genes across plant genomes. However, like genetic drift of deleterious alleles in populations, these putatively harmful *FBX* members could have largely expanded in few plant genomes if their activities are suppressed such as epigenetic suppression in Arabidopsis (Hua, 2021).

Our new purifying selection model of the plant *FBX* genes raised a new challenge in their functional genomic studies in plant genomes. Although many drifted members remain inactive, some, like drifted alleles, could restore their activities and thus play a role in plant adaptation. For example, out of 111 green plant genomes, *Kink Suppressed in BZR1-1D* (*KIB*) *1*/*2* and *EIN2 Targeting Protein* (*ETP*) *1/2* are two pairs of recently duplicated *FBX* genes that are only identified in 5 and 8 Brassicaceae species, respectively (Hua, 2021). However, they have been shown to promote degradation of Brassinosteroid (BR)-Insensitive 2 (BIN2) and EIN2, in BR and ethylene signaling pathways, respectively, in Arabidopsis (Qiao et al., 2009; Zhu et al., 2017). To effectively tackle the yet-unidentified pathways involving plant *FBX* genes, new approaches have to be utilized. In this work, we presented a machine learning approach to prioritizing the functionally active *FBX* members in Arabidopsis for our future functional genomic studies. This approach is based on analyses of multiple dimensional features of known *FBX* genes, with these attributes used to identify unknown candidates that likely play an active role in regulating plant growth and development. Such an approach is rare but striking in the field. We believe it could be also adapted into the functional genomic studies of many other UPS families, several of which are also composed of a large group of members.

## Materials and Methods

### Data Acquisition

The list of Arabidopsis *FBX* genes was selected based on two studies in Hua et al. (2011) and Hua (2021). Only the *FBX* genes predicted in both studies as well as annotated in Araport11 available at The Arabidopsis Information Resource (TAIR)^1^ were selected for further study. Based on the accession number, 11 characteristics of each *FBX* gene were collected from TAIR. These characteristics included (1) number of publications, (2) number of expression sequence tags (ESTs), (3) clones of complementary DNA (cDNAs), (4,5) number of introns and exons, (6) total number of transfer DNA (T-DNA) insertions in the genomic region of the *FBX* gene from 100 bp upstream of the transcription start site to the stop codon, and (7–11) the number of T-DNA insertions in five different regions of the *FBX* locus, which were defined as the 100 bp upstream of the transcription start site, the front and rear halves of the coding region, and the front and rear halves of the non-coding region.

The ratio of the number of non-synonymous substitutions per non-synonymous site (*K*_*a*_) to the number of synonymous substitutions per synonymous site (*K*_*s*_), *K*_*s*_ value, and neutral evolution feature of each *FBX* gene were retrieved from Hua et al. (2011). The protein-protein interaction domain information of each encoded FBX protein was obtained from Hua (2021). The subdomain families of each parental domain were combined and counted as the same group. The RNA-Seq expression data of each *FBX* gene was retrieved in batch at http://ipf.sustc.edu.cn/pub/athrna/ (Zhang et al., 2020).

### Multi-Dimensional Clustering Analysis

The resulting multi-characteristic data frame was subject to a k-means clustering analysis. The R package “ConsensusClusterPlus” was utilized to better determine the cluster number and clustering confidence, using the following settings: maxK = 9, reps = 1,000, pItem = 0.8, pFeature = 1, innerLinkage = “average,” finalLinkage = “average,” clusterAlg = “km,” distance = “Euclidean” (Monti et al., 2003; Wilkerson and Hayes, 2010).

### Sequence Alignment and Phylogenetic Analysis

The predicted FBXD sequences retrieved from each set of FBX proteins were compared and aligned with both MUSCLE (Edgar, 2004) and MAFFT (Katoh et al., 2019). The consensus alignment was resolved by trimming ambiguously aligned sites in both alignments using Trimal (-conthreshold 0.5) (Capella-Gutierrez et al., 2009). The resulting sequence alignment was used to conduct a maximum likelihood phylogenetic analysis in RAxML with a PROTGAMMAJTT substitution model (Stamatakis, 2014). The statistical significance was evaluated with 1,000 bootstrap replicates using a rapid bootstrap analysis.

### Supervised Machine Learning

Three machine learning approaches, including Artificial Neural Network (ANN) (Khan et al., 2001), Random Forest (RF) (Breiman, 1996, 2001; Diaz-Uriarte and Alvarez de Andres, 2006), and Support Vector Machine (SVM) (Vanitha et al., 2015), were adopted from the R packages, “neuralnet” (Fritsch et al., 2019), “randomForest” (Liaw and Wiener, 2002), and “e1071” (Meyer et al., 2020), respectively. After multiple runs, the settings for developing each machine learning model were optimized as follows: (1) we used two hidden layers with 10 and 2 nodes for ANN modeling; (2) a default setting except for “mtry = 4” was used in RF analysis; (3) for SVM learning models, we used the following settings: type = “C-classification”; kernel = “radial.”

All the machine learning predictions were performed on the same input dataset for 10 rounds with each round undergoing 1,000 times of resampling. In each resampling analysis, three different sets of samples, including training, validating, and testing samples, were selected from a total of 692 *FBX* genes with different levels of functional understanding up to date. From a pool of 41 well-studied and 123 out of 140 poorly studied *FBX* genes (1:3 ratio), we randomly selected 109 and 55 of them (2:1 ratio) as training and validating samples, respectively (Supplementary Script 1). The validating sample was used to examine prediction accuracy for well and poorly studied *FBX* genes based on the prediction model developed using the training sample. The testing sample contains the remaining 470 functionally unknown *FBX* genes that were examined and a second set of 41 known *FBX* genes serving as internal positive controls. The functionally active and inactive *FBX* genes predicted in 950 out of 1,000 times of resampling analysis were separately combined into two final prediction datasets. If an *FBX* gene was identified in 9 or greater from 10 rounds of predictions, it was considered a good candidate in each of the two final prediction datasets.

### Statistical Analysis by R

The statistical analyses were performed using in-house R scripts as described in Supplementary Scripts 1, 2 based on the processed data in Supplementary Data 1–3.

## Results

### Categorizing Arabidopsis *FBX* Genes

Based on our previous phylogenetic studies of *FBX* genes in 18 plant genomes (Hua et al., 2011), we retrieved the Arabidopsis members and searched the literature record of their functional studies at TAIR (see text footnote 1). Since our discovery on the genomic drift evolution of the *FBX* gene superfamily in plants (Hua et al., 2011), much effort has been made in the field to better understand the genomic and biochemical features of FBX proteins, which, in part, is evidenced by the update of five different subgroups of FBX Pfam-HMM profiles, including F-box, F-box-like, F-box-like_2, F-box_4, and F-box_5 (Pfam 32)^2^. To better predict the plant *FBX* genes in each genome, we recently developed a plant specific FBX HMM profile, named AO_FBX.hmm, based on 1,341 non-redundant FBXD sequences predicted in Arabidopsis and rice (Hua et al., 2011; Hua, 2021). Using these six FBX HMM profiles, we applied a Closing Target Trimming (CTT) high throughput superfamily annotation method and predicted in total 78,471 *FBX* genes in 111 plant species (Hua and Early, 2019; Hua, 2021). According to this new prediction, 14 previously predicted Arabidopsis *FBX* genes were not further analyzed in this work. In total, 696 *FBX* genes were selected for further analysis (Supplementary Data 1).

**FIGURE 1:**
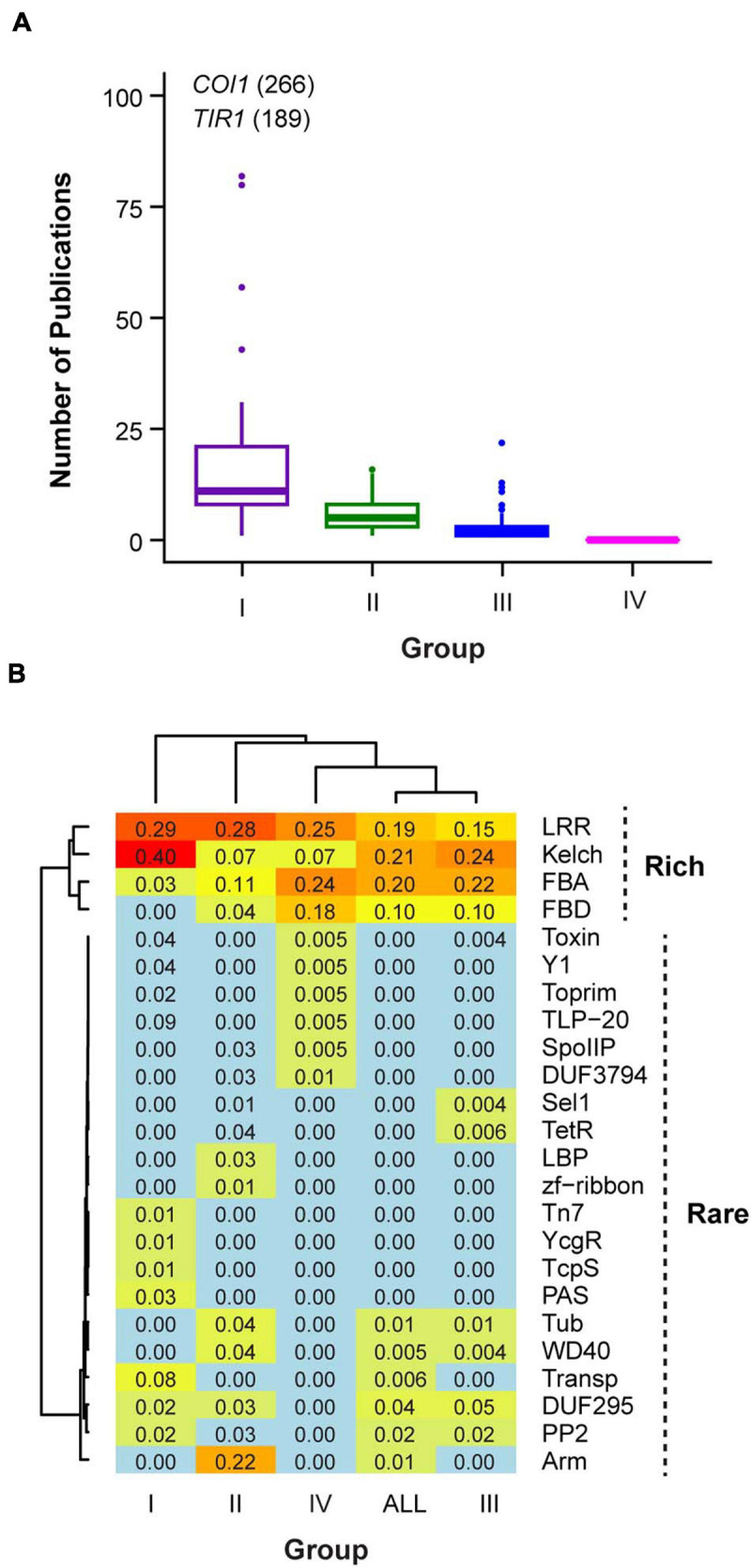
Categorizing four groups of Arabidopsis *FBX* genes. **(A)** Comparison of number of publications related to each *FBX* gene. **(B)** Differential frequencies of CTDs in each group of FBX proteins resulted in rich and rare groups of CTDs. The number in each grid shows the proportion of CTD-containing FBX proteins in each indicated group or the entire *FBX* family.

Through careful literature studies, we hypothesized that the functionality of an *FBX* gene is correlated with its number of publications because a functionally active gene is relatively easy to be identified and could be involved in multitude pathways that result in more publications. For example, *Transport Inhibitor Response 1* (*TIR1*) and *COronatine Insensitive 1* (*COI1*) are two *FBX* members that have more than 100 publications, which are consistent with their important roles as the receptors for auxin and jasmonic acid, respectively (Xie et al., 1998; Dharmasiri et al., 2005; Tan et al., 2007; Sheard et al., 2010). Hence, we categorized the total of 696 Arabidopsis *FBX* genes into four groups based on how well they have been studied up to date (Supplementary Data 1). If an *FBX* gene is well studied with its substrate also characterized, we considered it as a Group I member. In total 41 Group I *FBX* genes were identified (Supplementary Table 1). Importantly, the protein products of all Group I *FBX* genes have been demonstrated to interact with ASK1 protein (Supplementary Table 1), suggesting that ASK1 is a predominant Skp1 member in Arabidopsis as has been discovered in our previous studies (Hua and Gao, 2019; Yapa et al., 2020). There are also 41 *FBX* genes that have been phenotypically characterized with observable mutant phenotypes but without known substrates. We assigned them into Group II (Supplementary Data 1). Next generation sequencing technology has benefited a number of transcriptomic studies. If the expression of an *FBX* gene significantly responds to specific physiological and/or developmental processes, it could have been identified in these studies although its mutant phenotype and molecular mechanism are unknown. In total, 472 members have been reported to be significantly differentially expressed in 121 transcriptome-wide studies (retrieved from TAIR, see text footnote 1). We defined them as Group III *FBX* genes. The remaining 142 *FBX* genes that have never been reported in any work described above were combined as Group IV members. Not surprisingly, the number of publications varied significantly among these four groups (Figure 1A; *p* = 0.04 for Group I and Group II comparison and *p* = 0 for all the other pairwise comparisons, Kruskal-Wallis rank sum test followed by Dunn’s test with Benjamini-Hochberg multiple testing correction). While Group I *FBX* genes have 27 ± 50 (mean ± SD, hereafter the same) publications per member, each of the remaining *FBX* genes has 5.8 ± 3.8, 2.2 ± 1.9, and 0 publications in Groups II, III, and IV, respectively (Figure 1A).

### Sequence Comparison Between Known and Unknown *FBX* Genes

The specificity of an FBX protein is primarily determined by its C-terminal substrate recognition module (Zheng et al., 2002; Hua and Vierstra, 2011). To examine whether the differential functionalities of *FBX* genes are attributed to the C-terminal sequence variance of their encoded proteins, we annotated all known protein-protein interaction domains by searching the FBX protein sequences against the Pfam-A database (Pfam 32; see text footnote 2), which contained the HMM profiles for 17,933 protein-protein interaction domains (families). In total, 238 different domains were identified as putative C-terminal substrate binding domains (CTDs) (Supplementary Data 2).

By combining the top 10 abundant CTDs from each of the four groups and the entire set of FBX proteins (All), we identified in total 24 CTDs that are differentially represented. We clustered the CTDs according to their frequencies in each group. Interestingly, two distinct clusters of CTDs are resolved. While Leucine Rich Repeats (LRR), Kelch, FBX associated (FBA), and FBX binding (FBD) domains are clustered in a group that represents 70% of the total Arabidopsis FBX proteins, the remaining 20 CTDs are present in fewer FBX proteins except for Arm, which is only found in Group II (Figure 1B).

Among the four different groups of FBX proteins compared, Group I is significantly enriched with Kelch domains (Fisher’s exact test, *p* = 6.2e-4, 2.9e-2, and 6.3e-6 in comparison with Groups II, III, and IV, respectively). TIR1 protein domain (Transp-inhibit) and Arm domains are exclusively present in Groups I and II FBX proteins, respectively (Figure 1B). FBA and FBD seem to be enriched in the protein sequences encoded by *FBX* genes that are not well studied. They have the highest frequencies in Group IV followed by Group III FBX members. Some rare CTDs are also unique to Group III or IV groups. For example, Toxin, Y1, Toprim, TLP-20, SpoIIP, and DUF3794 are only found in Group IV FBX proteins and Sel1 and TetR are unique to Group III FBX proteins. It is yet unknown whether some of these rare CTDs resulted from the rapid sequence divergence process of recently duplicated *FBX* genes. It is known that recently duplicated *FBX* genes have high rates of non-synonymous mutations and frequent shifts of exon-intron boundaries (Xu et al., 2009; Hua et al., 2011). Such high rates of mutations may generate rare CTDs *de novo*. To support this hypothesis, we searched these rare CTDs in the entire predicted Arabidopsis proteome (in total 27,654 proteins). Among 30 protein sequences found to possess one of these CTDs, 13 also contain an FBXD, indicating an overrepresentation of FBX proteins in these rare CTD-containing proteins compared to the entire proteome (43% vs. 2.3%, Fisher’s exact test, *p* = 1.5e-13).

### Phylogenetic Comparison of Four Groups of *F-box* Genes

**FIGURE 2:**
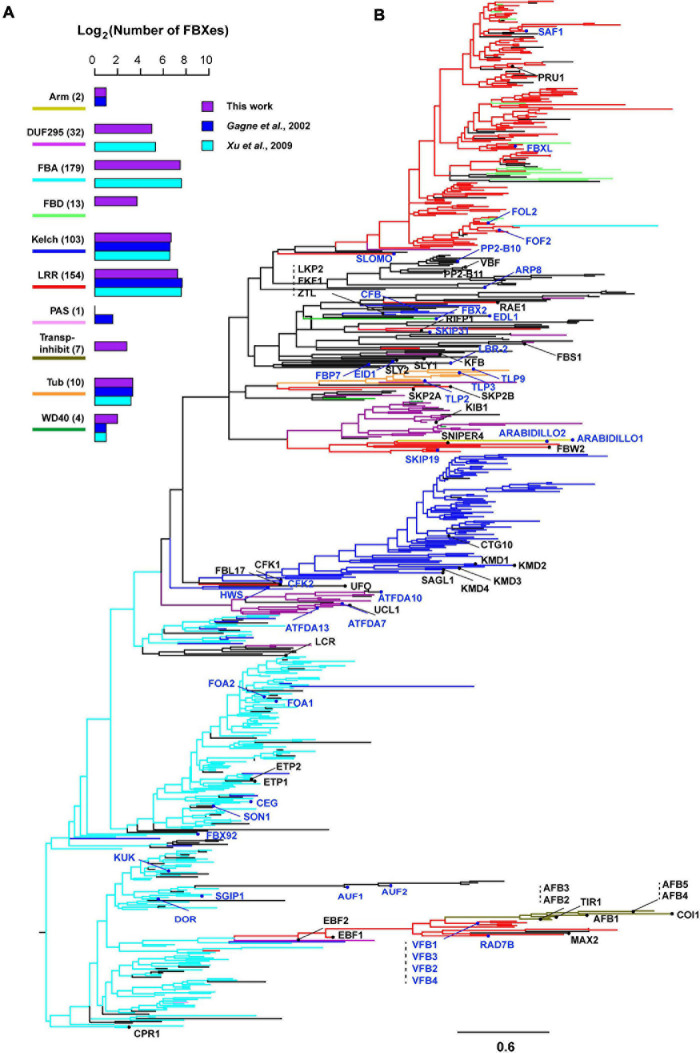
Phylogenetic distribution of four groups of Arabidopsis *FBX* genes. **(A)** Number comparison of FBX proteins containing top 10 CTDs in three different studies indicates slight prediction changes. The number shown in the parenthesis indicates the number of FBX proteins identified in this study. **(B)** A maximum likelihood phylogenetic analysis of 696 *FBX* genes based on their encoded FBXD sequences. The most abundant CTD in each FBX protein was identified and color coded as in **(A)** in the branches toward the node where it resides. The tree was generated using RAxML with a PROTGAMMAJTT substitution model. The known names of Groups I and II FBX proteins were labeled and highlighted with red and blue colors, respectively. Scale bar: amino acid changes per site. An expanded version with statistically significant bootstrap values can be found in Supplementary Figure 1.

Recently duplicated *FBX* genes are likely phylogenetically clustered together to form separate groups from ancient duplicates. Since ancient members may be under strong functional constrains for their long period of retention in the genome, we speculated that Groups I and II *FBX* genes may be clustered differently with the other two groups. To test this hypothesis, we retrieved the FBXD sequence of each FBX protein and carried out a maximum likelihood phylogenetic analysis. Although the majority of bootstrap values are too low to be statistically significant, the phylogenetic tree of 696 FBXD sequences can be approximately divided into FBA, Kelch, unknown, and LRR four large groups along with several small subfamilies, such as TIR1-containing Transp-Inhibit, TUB, WD40, DUF295, and FBD subfamilies, some of which (e.g., Transp-Inhibit, TUB, DUF295) are in general supported with a high bootstrap value (Figure 2A and Supplementary Figure 1). Therefore, the FBXD sequences were clustered in groups consistent with the CTD feature of an FBX protein, further suggesting a coevolutionary process between the FBXD and the CTD in an FBX protein sequence (Figure 2B; Gagne et al., 2002). However, surprisingly, the FBXD sequences encoded by Groups I and II genes are not completely clustered into isolated clades from those of Groups III and IV *FBX* genes. Although they are enriched in LRR, Kelch, and Arm domain encoding *FBX* genes, some also code a CTD that contains FBA and other unknown or rare domains (Figures 1B, 2B and Supplementary Figure 1), making the phylogenetic analysis difficult for predicting *FBX* members that are functionally active.

### An Unsupervised Clustering Approach to Finding Active *FBX* Genes

The wide distribution of known *FBX* genes in the phylogenetic tree found in this study and our recent finding about the purifying and dosage balancing selections on the *FBX* gene duplication process suggest two important evolutionary characteristics of Arabidopsis *FBX* genes. First, a significant number of *FBX* genes are functionally inactive and remain lineage/species-specific due to purifying/negative selection that prevents them from expanding across genomes. Second, functionally active *FBX* genes could arise from the lineage/species-specific group, such as *KIB 1/2* and *ETP1/2* in the *DUF295* and *FBA* groups, respectively (Figure 2B and Supplementary Figure 1). To effectively guide future functional genomic studies of the Arabidopsis *FBX* genes, we hypothesized that the functionally active *FBX* members share some common genomic, sequence, and transcriptomic features that may allow us to predict their relationship. Hence, we developed a multiple dimensional dataset that includes 27 characteristics of 692 Arabidopsis *FBX* genes (Supplementary Data 3). Four *FBX* genes, *AT1G24800* and *AT1G25055* from Group III and *AT5G36730* and *AT5G36820* from Group IV, were removed for further studies due to their lack of any data in a large collection of RNA-Seq expression dataset with 20,068 samples (Zhang et al., 2020).

**FIGURE 3:**
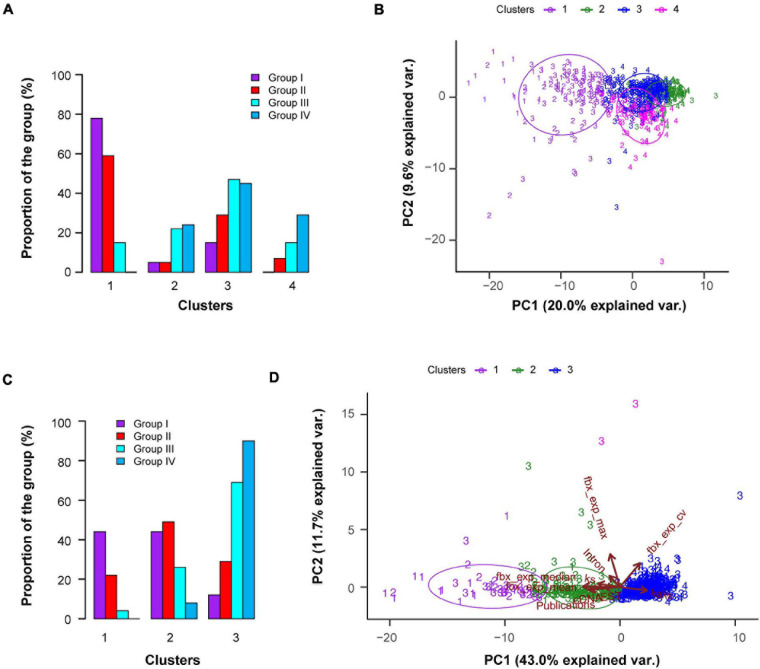
Unsupervised k-means clustering demonstrates distinct and overlapping features of four predefined groups of *FBX* genes. **(A)** Fraction distribution of four predefined groups of *FBX* genes as in Figure 1A in four k-means clusters classified based on 27 *FBX* characteristics shown in Supplementary Data 3. **(B)** A biplot showing the first two dimensions of a principle component analysis (PCA) of four groups of *FBX* genes. PC1 and 2 indicates the percentage of variance between individuals. Colored data points indicate the four clusters obtained from the analysis in **(A)** and the numbers indicate the four predefined groups of *FBX* genes. **(C)** Fraction distribution of four predefined groups of *FBX* genes in three k-means clusters as analyzed in **(A)** except for using 10 out of 27 *FBX* characteristics available in Supplementary Data 3. **(D)** Multivariate biplot of PCA analysis of four groups of *FBX* genes based on the same data set as in **(C)**. The contribution of the first two PCs explained 54.7% of the total variation. Data points are color coded and labeled as in **(B)**. Each arrow indicates the direction of the largest effect of the corresponding variable (characteristics) and the length of the arrow shows its influential strength. The angle between one pair of arrows reflects their correlations in the data set.

Based on this large data collection, we utilized a resampling-based unbiased k-means clustering method (Monti et al., 2003; Wilkerson and Hayes, 2010) to search for a potential list of genomic features that may be correlated with the functional activities of *FBX* genes. First, we analyzed the entire dataset to identify four k-means clusters (Figure 3A). Cluster 1 is significantly more enriched with Groups I and II *FBX* genes than with those from the other two groups. Cluster 3 seems to contain a similar proportion of *FBX* members from Groups II, III, and IV whereas the remaining two clusters (2 and 4) are enriched with Groups III and IV *FBX* genes. The differential clustering result of the four groups of *FBX* genes confirms the presence of distinct genomic and functional features among *FBX* genes. To further examine how well the four clusters of *FBX* genes were separated, we performed principal component analysis (PCA). Unfortunately, PC1 and PC2 only explained a mild proportion of the variance across 692 *FBX* genes which resulted in a large fraction of *FBX* genes that were overlapped among Clusters 2, 3, and 4 (Figure 3B). Hence, some vectors (characteristics) in the dataset disrupted the classification of *FBX* genes.

To better distinguish *FBX* genes with different functional activities, we performed multiple k-means clustering by selecting different number of vector combinations from the same dataset. We found three k-means clusters calculated based on 10 selected characteristics to better separate the four groups of *FBX* genes identified above (Figures 1, 3C,D). While Cluster 1 enriched Groups I and II *FBX* genes, the large fractions of Groups III and IV *FBX* genes were present in Cluster 3. Cluster 2 contains a significant proportion of Groups I, II, and III *FBX* genes. The PCA result demonstrated that 43.0 and 11.7% of the variance among the *FBX* genes could be explained by PC1 and PC2, respectively (Figure 3D). Among 10 vectors, mean and median expression, the number of complementary DNAs (cDNAs) and expression sequence tags, number of publications, and *K*_*s*_ values are positively correlated with the functional activities of *FBX* genes (i.e., more Groups I and II members). Conversely, the higher the expression coefficient variation (CV) and the *K*_*a*_/*K*_*s*_ value, the less active the *FBX* gene is (i.e., more Groups III and IV members). The maximum expression value and the number of introns seem not so distinguishable as the other vectors among *FBX* genes with different functional activities (Supplementary Data 3). Our previous study has discovered that the highest expression of many *FBX* genes could result from epigenomic programming regulation but not necessarily be related to its functional activity (Hua et al., 2013). The lack of correlation between the maximum expression value and the functional activity of an *FBX* gene further confirmed this notion.

### Ranking the Top Candidates of Unidentified Functionally Active *FBX* Genes by Neural Network Machine Learning

Clustering analysis found a significant number of Groups I and II *FBX* genes that were clustered together with the other two groups. For example, 15, 29, 47, and 46% of Groups I, II, III, and IV *FBX* genes, respectively, were clustered in Cluster 3 if we evaluated all 27 vectors (Figure 3A). When we used a better clustering data matrix, 44, 49, and 26% of Groups I, II, and III *FBX* genes, respectively, were found in Cluster 2. More intriguingly, only 9% of Group IV *FBX* genes were present in this cluster, suggesting that some Group III *FBX* genes could be also functionally active. Given that 41 Group I *FBX* genes are much better studied than any other group members and that 140 Group IV members have never been studied up to date (Figure 1A), we assigned them as functionally active and inactive members, respectively. Based on this prior condition, we sought to use a supervised machine learning approach to rank the functional activities of 470 Group III *FBX* genes whose functions are yet unknown.

Artificial neural network (ANN) is a machine learning algorithm that simulates the structure and behavior of human brain neurons (Khan et al., 2001). ANN applies a binary classification model to train and categorize complex patterns that are hidden in a large dataset. It operates an interconnected set of nodes with three kinds of layers, including input, hidden, and output layers, to make stepwise decisions (Greer and Khan, 2004). Since the data structure can change when external or internal data information flows through the network, it is suitable for analyzing non-linear interactions between dependent and independent variables (Pirooznia et al., 2008). Taking advantage of ANN decision analysis, we developed a novel bioinformatic pipeline to rank the activities of Arabidopsis *FBX* genes.

As described above, we treated Groups I and IV *FBX* genes as being functionally active and inactive, i.e., 1 and 0 for ANN analysis, respectively (Figure 1A, Supplementary Data 1, and Supplementary Script 1). In total, 41 Group I and 123 out of 140 Group IV (1:3 ratio) *FBX* genes were combined and randomly sampled into two datasets containing 109 and 55 *FBX* genes (2:1 ratio), which were used as training and validating samples, respectively (Supplementary Script 1). The validating sample was used to examine prediction accuracy and false discovery rate. The test sample includes 41 and 470 *FBX* genes from Groups II and III, respectively. Since Group II *FBX* genes have been phenotypically characterized with known mutant phenotypes, we further used this group of *FBX* genes as internal controls for examining the efficiency of our prediction. To increase the prediction confidence, we selected the consistent predictions from 9 or greater of 10 rounds of ANN analyses as our final result. In addition, we ran 1,000 times of resampling for each round of ANN analysis and only if an *FBX* gene was predicted in 950 out of 1,000 times of resampling would we consider it as a functionally active or inactive candidate in that round of analysis.

**FIGURE 4:**
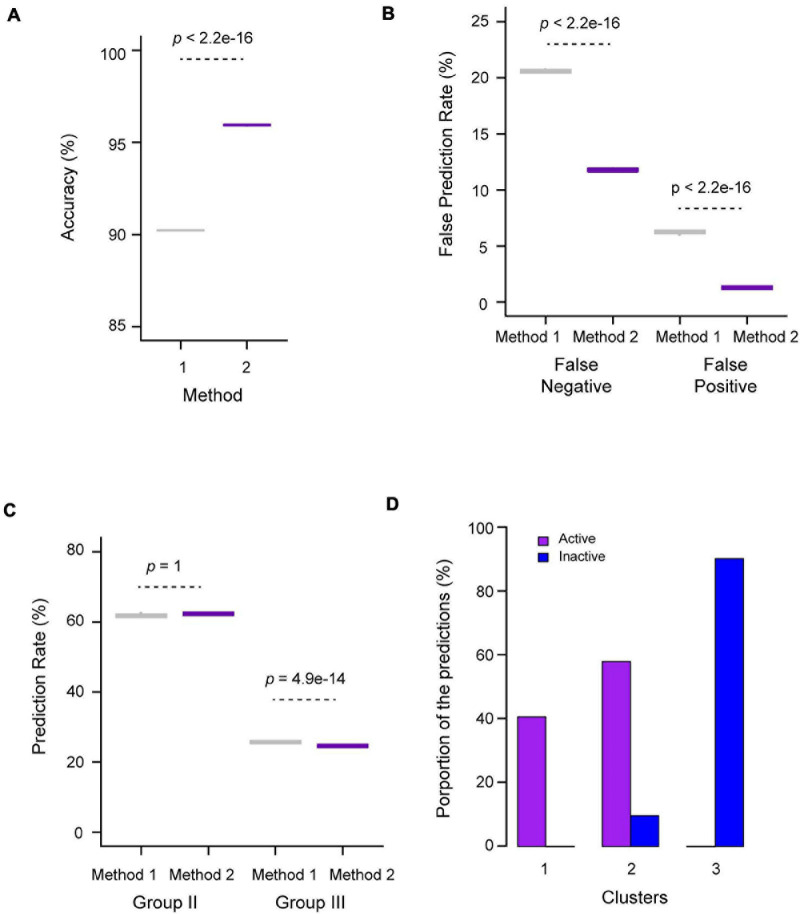
Performance evaluation of ANN prediction for functionally active and inactive *FBX* genes. **(A)** Prediction accuracy based on predefined Group I (active) and Group IV (inactive) validating *FBX* genes. Methods 1 and 2 utilized the dataset containing 27 and 10 variables, respectively, from Supplementary Data 3 as described in Figure 3. **(B)** False predication rates based on predefined Group I (active) and Group IV (inactive) testing *FBX* genes. Methods 1 and 2 are as in **(A)**. **(C)** Fractions of predicted active *FBX* genes in Groups II and III. Methods 1 and 2 are as in **(A)**. **(D)** Distribution of predicted active *FBX* genes in three k-means clusters obtained in Figures 3A–D. The number of *p*-values shown in **(A–C)** were calculated based on Welch two sample *t*-test.

Given the differential k-means clustering results from two datasets that contain 27 and 10 characteristics of the *FBX* genes (Figure 3), we also examined their influences on the ANN performance, which were designated as Methods 1 and 2, respectively. Interestingly, we observed an overall better prediction in Method 2 compared to Method 1. For example, on average, 96% of the validating samples (53 out of 55 *FBX* genes) were accurately predicted in Method 2 whereas Method 1 yielded 90.2% accuracy on this prediction (Figure 4A). Consequently, Method 1 resulted in 8.8 and 5.0% more false negative and false positive predictions, respectively, than Method 2 (Figure 4B). The high false prediction rate gave Method 1 to predict slightly more active *FBX* genes than Method 2 (Figure 4C). However, the goal of our machine learning is to find the best but not the highest number of candidates that could facilitate the finding of new *FBX* gene functions. Hence, similar to the unsupervised k-means clustering analysis, the shortened list of genomic and transcriptomic characteristics may better predict functionally active *FBX* genes. Consistently, compared to the k-means clustering result of the same dataset (10 characteristics, Figures 3C,D), the predicted functionally active *FBX* genes candidates are present in Clusters 1 and 2, while 90.2% of predicted functionally inactive *FBX* genes candidates are present in Cluster 3 (Figure 4D). Not surprisingly, Group II *FBX* genes (internal positive controls) were predicted to be significantly more enriched in the functionally active group than in the inactive group (28% vs. 1.6%, *p* = 2.4e-09, Fisher’s exact test; Supplementary Data 4).

### Verification of Unknown Active *FBX* Genes by Alternative Machine Learning Approaches

The prediction of functionally active and inactive *FBX* genes by ANN analysis is encouraging. Although both the validating sample (containing Group I and IV *FBX* genes that serve as active and inactive controls) and the internal controls (Group II) suggest that ANN has a good performance (Figure 4), we further developed multiple lines of evidence, including bioinformatic, phylogenetic, expression, and evolutionary comparisons, to confirm the prediction precision.

In addition to ANN machine learning, several additional approaches are also available for this objective (Pirooznia et al., 2008). For example, Random Forest (RF) utilizes classification trees for clustering variables through bootstrap aggregation and random selection for tree construction (Breiman, 1996, 2001; Diaz-Uriarte and Alvarez de Andres, 2006). Support Vector Machine (SVM) is another approach for variable clustering based on structural risk minimization (SRM) theory (Vanitha et al., 2015). Both RF and SVM have been widely applied for decision making upon input of a large dataset. Therefore, we also utilized these two machine learning approaches to predict functionally active and inactive *FBX* candidates based on the same dataset used for Method 2 of ANN prediction.

**FIGURE 5:**
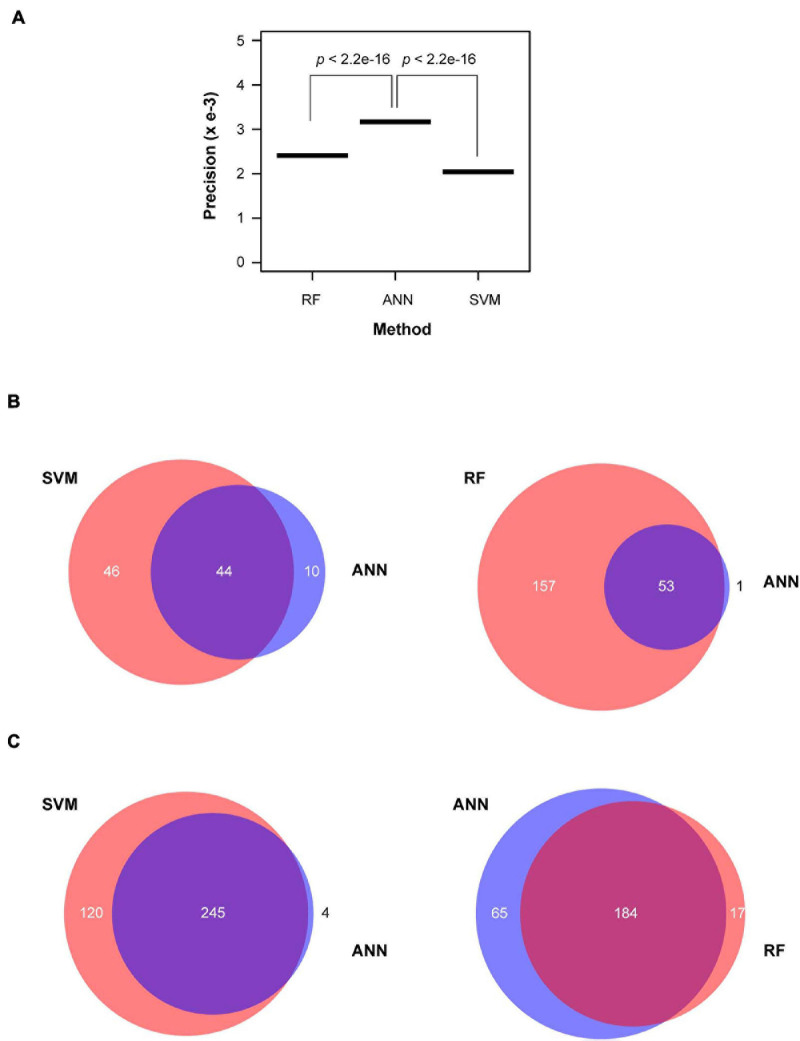
Performance comparison of ANN prediction with SVM and RF machine learning approaches. **(A)** Normalized prediction accuracy. The prediction accuracy calculated based on the same validating samples as described in Figure 4A was normalized by the total number of predicted active and inactive *FBX* genes. **(B)** A Venn diagram plotting showing the common and unique predictions of active *FBX* genes obtained from ANN with those obtained from SVM (left panel) and RF (right panel). **(C)** A Venn diagram plotting showing the common and unique predictions of inactive *FBX* genes obtained from ANN with those obtained from SVM (left panel) and RF (right panel).

The prediction accuracy from validating sample could be sensitive to the threshold applied in different algorithms. High prediction accuracy from the validating sample may sacrifice the prediction precision in test samples due to an unknown ratio of true and false members in the test samples. To better evaluate the performance of three machine learning approaches, we normalized the prediction accuracy from each validating sample by the total number of predicted functionally active and inactive *FBX* genes from the test sample. We defined this value as prediction precision. Interestingly, ANN outperformed both RF and SVM approaches (Figure 5A). More encouragingly, 44 and 53 out of 54 functionally active *FBX* genes predicted by ANN were also predicted by SVM and RF, respectively. However, the latter two methods yielded 1.7- and 3.9-fold more functionally active *FBX* genes than what ANN predicted (Figure 5B). Such a prediction is not very helpful for guiding future functional genomic studies. More *FBX* genes predicted can potentially weaken the priority of good candidates. In addition, ANN predicted a similar group of functionally inactive *FBX* genes as did RF although SVM predicted more members in this category (Figure 5C). Such variance can be neglected because the ultimate goal of this work is to guide the finding of new functions of functionally active *FBX* genes.

### Phylogenetic Verification of Unknown Active *FBX* Genes

The exclusive consistency in predicting the functionally active *FBX* genes among the three different machine learning approaches suggests that the predicted active and inactive *FBX* genes are significantly differentiated in their biological characteristics. Since ANN seemed to perform the best among the three approaches (Figure 5), we took the prediction of this approach (Method 2, Figure 4) for further verification. Because more inactive *FBX* genes were predicted (Figures 5B,C and Supplementary Data 4), we randomly sampled this dataset in order to keep the same number of active and inactive *FBX* genes for comparison. We examined how the predicted active and inactive members are phylogenetically related to the truly active *FBX* genes. Using the same approach as we constructed the phylogenetic tree of the entire *FBX* family (Figure 2B), we obtained a maximum likelihood tree that incorporates both predicted active and inactive members and Group I *FBX* genes. For better comparison, the tree has been rooted to EIN3-Binding F-box protein 1 (EBF1).

**FIGURE 6:**
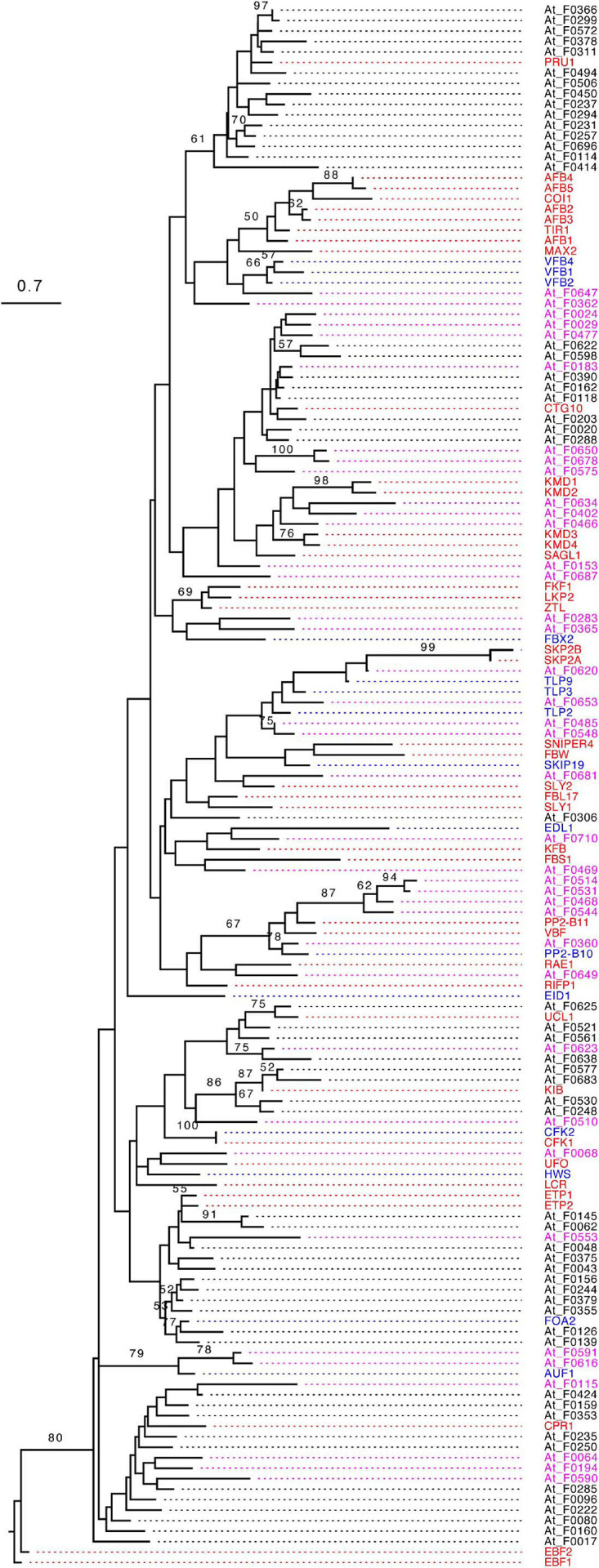
Phylogenetic comparison of predicted active and inactive *FBX* genes with known Group I members. The phylogenetic tree was constructed as in Figure 2B except that the statistical significance equal to or greater than 50% of 1,000 times of bootstrap resampling is indicated in each corresponding node. The known names of Groups I and II FBX proteins were labeled and highlighted with red and blue colors, respectively. The identification names [described in Hua et al. (2011)] of predicted functionally active and inactive Group III FBX proteins were highlighted with magenta and black colors, respectively. Size bar: amino acid changes per site.

Surprisingly, we found that all the members were clustered with Group I FBX proteins in one single clade with strong statistical significance (Figure 6). Since all Group I FBX proteins are known to interact with ASK1, the monophyletic relationship of both unknown active and inactive FBX proteins with known active Group I FBX proteins further argues that many, if not all, Arabidopsis FBX proteins bind to ASK1. However, the distribution of predicted functionally active and inactive FBX proteins shows distinct phylogenetic patterns in relation to Group I FBX proteins. While predicted active FBX members intermingle with Group I FBX members in forming multiple mixed subclades, the predicted inactive members are in general clustered together (Figure 6). Hence, we concluded that the predicted functionally active *FBX* members are more phylogenetically related to Group I *FBX* genes than inactive ones.

### Distinct Genomic and Transcriptomic Features Between Active and Inactive *FBX* Genes

To further demonstrate our prediction precision biologically, we compared the predicted functionally active and inactive *FBX* members with Group I *FBX* genes at both expression variance and evolutionary constraint levels.

**FIGURE 7:**
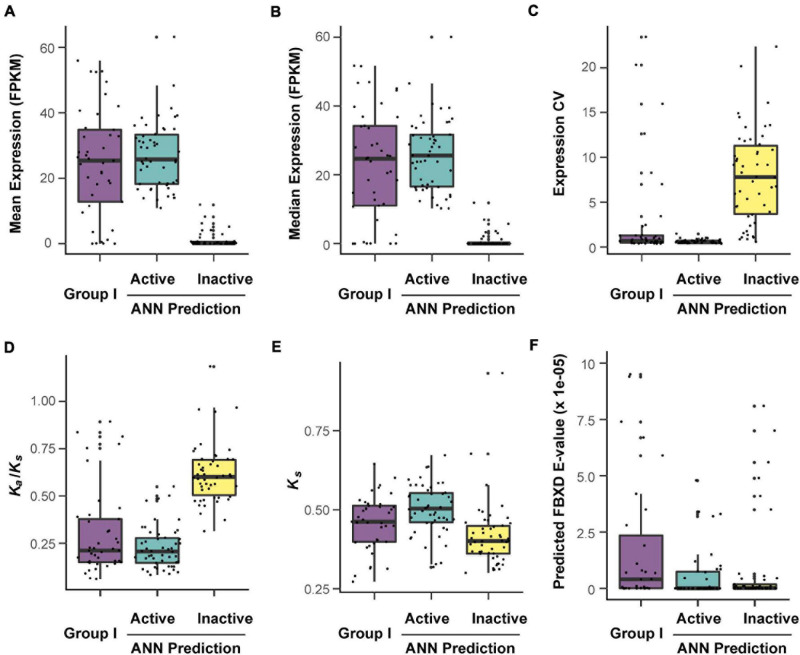
Verification of predicted active and inactive *FBX* genes according to their distinct expression and evolutionary features. The corresponding values of *FBX* genes were retrieved from Supplementary Data 3 and plotted against each other in the indicated groups. **(A–C)** Expression comparison of three indicated groups of *FBX* genes in 20,068 RNA-Seq samples (Zhang et al., 2020). **(A)** Mean expression per gene; **(B)** median expression per gene; **(C)** expression coefficient of variation (CV) per gene. **(D,E)** Distinction of evolutionary features among the indicated groups. **(F)** Comparison of predicted FBXD e-values among the indicated groups.

While no dramatic expression variance can be observed between Group I and the predicted functionally active *FBX* genes, both groups have extremely higher mean and median expression values than the predicted inactive *FBX* genes. Not surprisingly, the expression coefficient variance (CV) of predicted inactive *FBX* genes is significantly higher than the other two groups due to their extremely low mean expression values (Figures 7A–C; *p* = 0 for comparisons of Group I or predicted active *FBX* genes with inactive *FBX* genes, Kruskal-Wallis rank sum test followed by Dunn’s test with Benjamini-Hochberg multiple testing correction). Such dramatic expression variance between functionally active and inactive members suggests a good prediction precision of our dataset.

We further examined the difference of evolutionary constraints among these three groups. Functionally inactive genes are not always under strong evolutionary constraints and many could experience neutral changes, which result in high *K*_*a*_/*K*_*s*_ ratios. When plotted with the *K*_*a*_/*K*_*s*_ values of the *FBX* genes in three groups, the predicted group of functionally inactive *FBX* members showed an average of 0.62 ± 0.16 *K*_*a*_/*K*_*s*_ value, significantly higher than 0.23 ± 0.11 and 0.30 ± 0.23, respectively, for the *K*_*a*_/*K*_*s*_ values of the predicted functionally active *FBX* genes and Group I members. The *K*_*a*_/*K*_*s*_ values of the latter two groups were not statistically significantly different (Figure 7D). *K*_*s*_ values could be used to indirectly indicate the age and types of a gene duplicate. The higher the *K*_*s*_ the more likely the gene duplicate resulted from an ancient whole genome duplication event, which is in general highly constrained (Li et al., 2016; Hua, 2021). Hence, we also compared the *K*_*s*_ differences among the three groups (Figure 7E). Surprisingly, we found that the predicted functionally active *FBX* genes had the highest *K*_*s*_ values followed by Group I *FBX* genes. This result further concluded the strong functional constraints of our predicted active *FBX* genes. The low *K*_*s*_ values of the predicted inactive *FBX* members are consistent with their weak functionality in Arabidopsis.

Previous genomic studies on the *FBX* genes often applied a Pfam search e-value to predict the presence of an FBXD in its encoded protein sequence. However, we have argued a potential drawback of this method in finding most, if not all, *FBX* genes in genomes in our previous studies through comparing our result with those from other research groups (Hua et al., 2011). For example, several well-studied Group I FBX proteins have high e-values. To provide an additional line of evidence, we evaluate the predicted FBXD e-values among the three groups. We found that the FBXD e-values of predicted functionally inactive *FBX* members were significantly lower than Group I (Figure 7F; *p* = 0.007; Kruskal-Wallis rank sum test followed by Dunn’s test with Benjamini-Hochberg multiple testing correction). Although we cannot rule out the possibility of physical interaction between an encoded inactive FBX protein with Skp1, the significantly higher e-values of Group I FBX proteins further suggested that the e-value cannot be used as an effective criterion for predicting a functionally active *FBX* gene in genomes (Hua et al., 2011).

## Discussion

The plant *FBX* gene superfamily is arguably one of the largest, yet also largely unexplored, group of protein-coding genes. Although the past two decades of functional genomic studies in the model plant, Arabidopsis, have revealed a wide range of F-box protein functions, only 10% of the total ∼800 members have been genetically characterized (Supplementary Data 1; Hua et al., 2011, 2013; Hua, 2021). Making it even more challenging, the F-box proteins with known molecular mechanism and ubiquitylation substrates have been only about 5% of the family up to date (Supplementary Table 1). Not only the difficulties in proteomic identification of short-lived and low abundant FBX substrates but also the unique evolutionary processes made it extremely challenging to characterize the biological roles of *FBX* genes. In addition to our previous discovery showing the epigenomic suppression of a large set of Arabidopsis *FBX* genes (Hua et al., 2013), we recently proposed a novel evolutionary mechanism involving the *FBX* gene superfamily in 111 plant genomes (Hua, 2021). The study from this large group of plant species uncovered both purifying and dosage balancing selections that apply on different groups of plant *FBX* genes. While many inactive ones remain lineage/species-specific by strong purifying selection against their expansion in plant genomes, those active ones are under balancing selections whose copy numbers in a genome are determined by the pool of substrates. Such dual evolutionary processes may give rise to the unprecedented challenges in the functional genomic studies of the plant *FBX* genes.

Although evolutionary studies may help uncover a core group of plant FBX proteins, a significant proportion of the remaining lineage or even species-specific members could activate and restore an adaptive role for plant survival (Hua, 2021). Such members are hard to discover through evolutionary comparative studies. Fortunately, in part thanks to the advancement of next generation sequencing technologies, a tremendous amount of genomic and transcriptomic data has been accumulated up to date particularly in Arabidopsis. For example, we were only able to detect expression data for 330 Arabidopsis *FBX* gene in 4,933 microarrays available for Col-0 in the NASCArrays in 2013 (Hua et al., 2013). However, in this study, we found the expression data for 692 *FBX* genes in 20,068 RNA-Seq samples from Arabidopsis (Zhang et al., 2020), which significantly benefited us to decipher the expression variance of different groups of *FBX* genes.

Since some Group I *FBX* genes (well-studied) have a lower expression level than functionally inactive members, it would be challenging to identify them from functionally inactive members based on expression data (Figures 7A,B). To compare the relationship of *FBX* members with variant functionalities, one idea is to integrate their genomic, transcriptomic, sequence structure, and evolutionary features as many as possible. In this work, we collected 27 different types of *FBX* characteristics including number of publications, which served as indirect evidence of their activities (Supplementary Data 1, 3). Unsupervised k-means clustering was able to identify three or four separated clusters (Figure 3). However, due to significant overlaps among several clusters, such a clustering approach is not able to rank or prioritize the *FBX* gene members based on their functional activities. Fortunately, the development of multiple supervised machine learning algorithms in the science community allowed us to adapt them for our studies. The excellent prediction precision of ANN analysis is demonstrated by multiple lines of evidence in this study. First, it yielded ∼96% accuracy in predicting the predefined activities of a validating dataset (Figure 4A). Second, 98% of its predicted active *FBX* genes were also predicted by the other two machine learning approaches including SVM and RF, which were based on different algorithms (Figure 5B). Third, the *FBX* genes with known mutant phenotypes (Group II members in the test sample) were successfully predicted to be overrepresented in the functionally active group (Supplementary Data 4). Fourth, the predicted functionally active and inactive *FBX* genes demonstrated striking difference in phylogenetic relationship with known active *FBX* members (Figure 6). Fifth, both expression and evolutionary selection data further suggested that the predicted functionally active members were most likely active (Figure 7).

We believe that our approach in prioritizing the functionally active *FBX* members for future functional genomic studies is innovative. Such an approach can be routinely and iteratively applied to fine tune the best list of candidates based on what we know from the prior data. Although it seems that the more data the better, our study found that reducing some characteristics yielded a better classification of *FBX* genes in both k-means clustering and ANN machine learning (Figures 3, 4). Hence, irrelevant variables in the data matrix could impact the prediction accuracy by complicating calculation. Considering the enormous size of the plant UPS and its yet largely unknown substrates, developing machine learning approach-based artificial intelligent studies could effectively assist the discovery of new mechanisms in this system. However, the ultimate finding still relies on more effective and high throughput omics analyses in conjunction with individual fine-tuning work in both genetic and biochemical studies. The 50% of known *FBX* members lacking strong biochemical evidence (Group II) reflects the importance of developing this type of study in the field.

## Data Availability Statement

The original contributions presented in the study are included in the article/Supplementary Material, further inquiries can be directed to the corresponding author/s.

## Author Contributions

ZH conceived of the study, performed data analysis, wrote the manuscript, and gave final approval of the version to be published. YL drafted the machine learning approaches and assisted data analysis. YL and MMY assisted manuscript writing. All authors made substantial contributions to data acquisition, to interpretation and modification of the data, were involved in manuscript revisions, and read and approved the final manuscript.

## Conflict of Interest

The authors declare that the research was conducted in the absence of any commercial or financial relationships that could be construed as a potential conflict of interest.

## Footnotes

1 https://www.arabidopsis.org

2 https://pfam.xfam.org
