# Supplementary material for: A Machine Learning Approach to Prioritizing Functionally Active F-box Members in Arabidopsis thaliana
Source: Front Plant Sci. 2021 May 28;12:639253. doi: 10.3389/fpls.2021.639253 (PMC8192846; doi:10.3389/fpls.2021.639253)
Supplement: Supplementary file 1 [file Table_1.DOCX]

**Table S1.** List of Group 1 ASK1-interacting FBX proteins with known substrates

| ID | Accession | Name | Function | Substrate | Reference |
| --- | --- | --- | --- | --- | --- |
| At_F0430 | AT4G19330 | CTG10 | seed germination | PIF1 | (Majee et al., 2017;Majee et al., 2018) |
| At_F0267 | AT1G30950 | UFO | Floral organ development | LFY | (Samach et al., 1999) |
| At_F0313 | AT1G27340 | LCR | Leaf curling response | MLP | (Litholdo et al., 2016) |
| At_F0707 | AT1G65740 | UCL1 | Curly leaf | CLF | (Jeong et al., 2011;Jeong et al., 2015) |
| At_F0505 | AT4G08980 | FBW2 | MiRNA biogenesis | AGO1 | (Earley et al., 2010) |
| At_F0220 | AT5G42350 | CFK1 | Epigenetic regulation | DRM2 | (Chen et al., 2020) |
| At_F0474 | AT5G57360 | ZTL | Circadian rhythm | TOC1, PRR5 | (Más et al., 2003) (Kiba et al., 2007) |
| At_F0542 | AT2G18915 | LKP2 | Circadian rhythm | TOC1,  PRR5 | (Baudry et al., 2010) |
| At_F0585 | AT1G68050 | FKF1 | Circadian rhythm; flower time | CO/ DELLA | (Lee et al., 2017) (Yan et al., 2020) |
| At_F0508 | AT1G21410 | SKP2A | cell division | E2Fc | (del Pozo et al., 2002) |
| At_F0516 | AT1G77000 | SKP2B | Cell division;  Lateral root development | E2Fc | (del Pozo et al., 2002;Manzano et al., 2012) |
| At_F0563 | AT3G54650 | FBL17 | Cell cycle regulation | KRPs | (Noir et al., 2015) |
| At_F0586 | AT3G62980 | TIR1 | Auxin signaling | AUX/IAA | (Dharmasiri et al., 2005a;Dharmasiri et al., 2005b;Scheitz et al., 2013) |
| At_F0570 | AT4G03190 | AFB1 | Auxin signaling | AUX/IAA | (Dharmasiri et al., 2005b) |
| At_F0626 | AT3G26810 | AFB2 | Auxin signaling | AUX/IAA | (Dharmasiri et al., 2005b) |
| At_F0628 | AT1G12820 | AFB3 | Auxin signaling | AUX/IAA | (Dharmasiri et al., 2005b) |
| At_F0669 | AT4G24390 | AFB4 | Auxin signaling | AUX/IAA | (Prigge et al., 2016) |
| At_F0662 | AT5G49980 | AFB5 | Auxin signaling | AUX/IAA | (Prigge et al., 2016) |
| At_F0269 | AT1G80110 | PP2-B11 | ABA signaling | SnRK2.3 | (Cheng et al., 2017) |
| At_F0641 | AT3G26000 | RIFP1 | ABA signaling | RCAR3 | (Li et al., 2016) |
| At_F0549 | AT4G12810 | KIB1 | Brassinosteroid response | BIN2 | (Zhu et al., 2017) |
| At_F0564 | AT1G80440 | KMD1 | Cytokinin response; Phenylpropanoid biosynthesis | ARRS;  PAL1 | (Zhang et al., 2013) |
| At_F0656 | AT1G15670 | KMD2 | Cytokinin response; Phenylpropanoid pathway | ARRS;  PAL1 | (Kim et al., 2013;Zhang et al., 2013) |
| At_F0354 | AT2G44130 | KMD3 | Cytokinin response; Phenylpropanoid biosynthesis;  UV-B Stress response | ARRS;  PAL1 | (Zhang et al., 2015) |
| At_F0467 | AT3G59940 | KMD4 | Cytokinin response; Phenylpropanoid biosynthesis;  UV-B Stress response | ARRS;  PAL1 | (Zhang et al., 2015) |
| At_F0416 | AT2G25490 | EBF1 | Ethelene signaling | EIN3;  PIF3 | (Guo and Ecker, 2003;Potuschak et al., 2003;Gagne et al., 2004;Dong et al., 2017;Vaseva et al., 2018) |
| At_F0442 | AT5G25350 | EBF2 | Ethelene signaling | EIN3  PIF3 | (Guo and Ecker, 2003;Potuschak et al., 2003;Gagne et al., 2004;Dong et al., 2017;Vaseva et al., 2018) |
| At_F0158 | AT3G18980 | ETP1 | Ethelene response | EIN2 | (Qiao et al., 2009) |
| At_F0125 | AT3G18910 | ETP2 | Ethelene response | EIN2 | (Qiao et al., 2009) |
| At_F0234 | AT4G24210 | SLY1 | GA signaling | DELLA | (Nelson and Steber, 2017) |

| At_F0297 | AT5G48170 | SLY2 | GA signaling | DELLA | (Ariizumi et al., 2011) |
| --- | --- | --- | --- | --- | --- |
| At_F0705 | AT2G39940 | COI1 | Jasmonic acid signaling | JAZ | (Xu et al., 2002) |
| At_F0676 | AT2G42620 | MAX2 | Strigolactone and karrikin signaling | SMXL | (Wang et al., 2015) |
| At_F0500 | AT1G23390 | KFB | Phenylpropanoid biosynthesis | CHS | (Zhang et al., 2017) |
| At_F0445 | AT1G55270 | SAGL1 | phenylpropanoid biosynthesis | PAL1 | (Kim et al., 2019;Yu et al., 2019) |
| At_F0397 | AT4G12560 | CPR1 | Plant immunity | SNC1/ RPS2 | (Cheng et al., 2011) |
| At_F0538 | AT3G48880 | SNIPER4 | Immune response | MUSE | (Huang et al., 2018) |
| At_F0646 | AT1G56250 | VBF | Agrobacterium transformation | VIP1 | (Zaltsman et al., 2010) |
| At_F0168 | AT3G42770 | PRU1 | Low phosphate stress | WRKY6 | (Ye et al., 2018) |
| At_F0665 | AT5G01720 | RAE1 | Aluminum resistance | STOP1 | (Zhang et al., 2019) |
| At_F0674 | AT1G61340 | FBS1 | Biotic and abiotic stress responses | 14-3-3 | (Sepúlveda-García and Rocha-Sosa, 2012) |

(Continued)

**References**

Ariizumi, T., Lawrence, P.K., and Steber, C.M. (2011). The role of two f-box proteins, SLEEPY1 and SNEEZY, in Arabidopsis gibberellin signaling. *Plant Physiol* 155**,** 765-775.

Baudry, A., Ito, S., Song, Y.H., Strait, A.A., Kiba, T., Lu, S., Henriques, R., Pruneda-Paz, J.L., Chua, N.H., Tobin, E.M., Kay, S.A., and Imaizumi, T. (2010). F-box proteins FKF1 and LKP2 act in concert with ZEITLUPE to control Arabidopsis clock progression. *Plant Cell* 22**,** 606-622.

Chen, J., Jiang, J., Liu, J., Qian, S., Song, J., Kabara, R., Delo, I., Serino, G., Liu, F., Hua, Z., and Zhong, X. (2020). F-box protein CFK1 interacts with and degrades de novo DNA methyltransferase in Arabidopsis. *New Phytol* (in press).

Cheng, C., Wang, Z., Ren, Z., Zhi, L., Yao, B., Su, C., Liu, L., and Li, X. (2017). SCF^AtPP2-B11^ modulates ABA signaling by facilitating SnRK2.3 degradation in *Arabidopsis thaliana*. *PLoS Genet* 13**,** e1006947.

Cheng, Y.T., Li, Y., Huang, S., Huang, Y., Dong, X., Zhang, Y., and Li, X. (2011). Stability of plant immune-receptor resistance proteins is controlled by SKP1-CULLIN1-F-box (SCF)-mediated protein degradation. *Proc Natl Acad Sci U S A* 108**,** 14694-14699.

Del Pozo, J.C., Boniotti, M.B., and Gutierrez, C. (2002). Arabidopsis E2Fc functions in cell division and is degraded by the ubiquitin-SCF^AtSKP2^ pathway in response to light. *Plant Cell* 14**,** 3057-3071.

Dharmasiri, N., Dharmasiri, S., and Estelle, M. (2005a). The F-box protein TIR1 is an auxin receptor. *Nature* 435**,** 441-445.

Dharmasiri, N., Dharmasiri, S., Weijers, D., Lechner, E., Yamada, M., Hobbie, L., Ehrismann, J.S., Jürgens, G., and Estelle, M. (2005b). Plant development is regulated by a family of auxin receptor F box proteins. *Dev Cell* 9**,** 109-119.

Dong, J., Ni, W., Yu, R., Deng, X.W., Chen, H., and Wei, N. (2017). Light-dependent degradation of PIF3 by SCF^EBF1/2^ promotes a photomorphogenic response in Arabidopsis. *Curr Biol* 27**,** 2420-2430.e2426.

Earley, K., Smith, M.R., Weber, R., Gregory, B.D., and Poethig, R.S. (2010). An endogenous F-box protein regulates ARGONAUTE1 in *Arabidopsis thaliana*. *Silence* 1**,** 15.

Gagne, J.M., Smalle, J., Gingerich, D.J., Walker, J.M., Yoo, S.D., Yanagisawa, S., and Vierstra, R.D. (2004). Arabidopsis EIN3-binding F-box 1 and 2 form ubiquitin-protein ligases that repress ethylene action and promote growth by directing EIN3 degradation. *Proc Natl Acad Sci U S A* 101**,** 6803-6808.

Guo, H., and Ecker, J.R. (2003). Plant responses to ethylene gas are mediated by SCF^EBF1/EBF2^-dependent proteolysis of EIN3 transcription factor. *Cell* 115**,** 667-677.

Huang, J., Zhu, C., and Li, X. (2018). SCF^SNIPER4^ controls the turnover of two redundant TRAF proteins in plant immunity. *Plant J* 95**,** 504-515.

Jeong, C.W., Park, G.T., Yun, H., Hsieh, T.F., Choi, Y.D., Choi, Y., and Lee, J.S. (2015). Control of paternally expressed imprinted *UPWARD CURLY LEAF1*, a gene encoding an F-Box protein that regulates CURLY LEAF polycomb protein, in the Arabidopsis Endosperm. *PLoS One* 10**,** e0117431.

Jeong, C.W., Roh, H., Dang, T.V., Choi, Y.D., Fischer, R.L., Lee, J.S., and Choi, Y. (2011). An E3 ligase complex regulates SET-domain polycomb group protein activity in *Arabidopsis thaliana*. *Proc Natl Acad Sci U S A* 108**,** 8036-8041.

Kiba, T., Henriques, R., Sakakibara, H., and Chua, N.H. (2007). Targeted degradation of PSEUDO-RESPONSE REGULATOR5 by an SCF^ZTL^ complex regulates clock function and photomorphogenesis in *Arabidopsis thaliana*. *Plant Cell* 19**,** 2516-2530.

Kim, H., Yu, S.I., Jung, S.H., Lee, B.H., and Suh, M.C. (2019). The F-Box protein SAGL1 and ECERIFERUM3 regulate cuticular wax biosynthesis in response to changes in humidity in Arabidopsis. *Plant Cell* 31**,** 2223-2240.

Kim, H.J., Chiang, Y.H., Kieber, J.J., and Schaller, G.E. (2013). SCF^KMD^ controls cytokinin signaling by regulating the degradation of type-B response regulators. *Proc Natl Acad Sci U S A* 110**,** 10028-10033.

Lee, B.-D., Kim, M.R., Kang, M.-Y., Cha, J.-Y., Han, S.-H., Nawkar, G.M., Sakuraba, Y., Lee, S.Y., Imaizumi, T., Mcclung, C.R., Kim, W.-Y., and Paek, N.-C. (2017). The F-box protein FKF1 inhibits dimerization of COP1 in the control of photoperiodic flowering. *Nature Communications* 8**,** 2259.

Li, Y., Zhang, L., Li, D., Liu, Z., Wang, J., Li, X., and Yang, Y. (2016). The Arabidopsis F-box E3 ligase RIFP1 plays a negative role in abscisic acid signalling by facilitating ABA receptor RCAR3 degradation. *Plant Cell Environ* 39**,** 571-582.

Litholdo, C.G., Jr., Parker, B.L., Eamens, A.L., Larsen, M.R., Cordwell, S.J., and Waterhouse, P.M. (2016). Proteomic identification of putative MicroRNA394 target genes in *Arabidopsis thaliana* identifies major latex protein family members critical for normal development. *Mol Cell Proteomics* 15**,** 2033-2047.

Majee, M., Kumar, S., Kathare, P.K., Wu, S., Gingerich, D., Nayak, N.R., Salaita, L., Dinkins, R., Martin, K., Goodin, M., Dirk, L.M.A., Lloyd, T.D., Zhu, L., Chappell, J., Hunt, A.G., Vierstra, R., Huq, E., and Downie, A.B. (2018). KELCH F-BOX protein positively influences Arabidopsis seed germination by targeting PHYTOCHROME-INTERACTING FACTOR1. *Proc Natl Acad Sci U S A* 115**,** E4120-E4129.

Majee, M., Wu, S., Salaita, L., Gingerich, D., Dirk, L.M.A., Chappell, J., Hunt, A.G., Vierstra, R., and Downie, A.B. (2017). A misannotated locus positively influencing Arabidopsis seed germination is deconvoluted using multiple methods, including surrogate splicing. *Plant Gene* 10**,** 74-85.

Manzano, C., Ramirez-Parra, E., Casimiro, I., Otero, S., Desvoyes, B., De Rybel, B., Beeckman, T., Casero, P., Gutierrez, C., and J, C.D.P. (2012). Auxin and epigenetic regulation of SKP2B, an F-box that represses lateral root formation. *Plant Physiol* 160**,** 749-762.

Más, P., Kim, W.Y., Somers, D.E., and Kay, S.A. (2003). Targeted degradation of TOC1 by ZTL modulates circadian function in Arabidopsis thaliana. *Nature* 426**,** 567-570.

Nelson, S.K., and Steber, C.M. (2017). Transcriptional mechanisms associated with seed dormancy and dormancy loss in the gibberellin-insensitive *sly1-2* mutant of *Arabidopsis thaliana*. *PLoS One* 12**,** e0179143.

Noir, S., Marrocco, K., Masoud, K., Thomann, A., Gusti, A., Bitrian, M., Schnittger, A., and Genschik, P. (2015). The control of *Arabidopsis thaliana* growth by cell proliferation and endoreplication requires the F-Box protein FBL17. *Plant Cell* 27**,** 1461-1476.

Potuschak, T., Lechner, E., Parmentier, Y., Yanagisawa, S., Grava, S., Koncz, C., and Genschik, P. (2003). EIN3-dependent regulation of plant ethylene hormone signaling by two Arabidopsis F-box proteins: EBF1 and EBF2. *Cell* 115**,** 679-689.

Prigge, M.J., Greenham, K., Zhang, Y., Santner, A., Castillejo, C., Mutka, A.M., O'malley, R.C., Ecker, J.R., Kunkel, B.N., and Estelle, M. (2016). The Arabidopsis auxin receptor F-Box proteins AFB4 and AFB5 are required for response to the synthetic auxin picloram. *G3 (Bethesda)* 6**,** 1383-1390.

Qiao, H., Chang, K.N., Yazaki, J., and Ecker, J.R. (2009). Interplay between ethylene, ETP1/ETP2 F-box proteins, and degradation of EIN2 triggers ethylene responses in Arabidopsis. *Genes Dev* 23**,** 512-521.

Samach, A., Klenz, J.E., Kohalmi, S.E., Risseeuw, E., Haughn, G.W., and Crosby, W.L. (1999). The UNUSUAL FLORAL ORGANS gene of Arabidopsis thaliana is an F-box protein required for normal patterning and growth in the floral meristem. *Plant J* 20**,** 433-445.

Scheitz, K., Lüthen, H., and Schenck, D. (2013). Rapid auxin-induced root growth inhibition requires the TIR and AFB auxin receptors. *Planta* 238**,** 1171-1176.

Sepúlveda-García, E., and Rocha-Sosa, M. (2012). The Arabidopsis F-box protein AtFBS1 interacts with 14-3-3 proteins. *Plant Sci* 195**,** 36-47.

Vaseva, Ii, Qudeimat, E., Potuschak, T., Du, Y., Genschik, P., Vandenbussche, F., and Van Der Straeten, D. (2018). The plant hormone ethylene restricts Arabidopsis growth via the epidermis. *Proc Natl Acad Sci U S A* 115**,** E4130-e4139.

Wang, L., Wang, B., Jiang, L., Liu, X., Li, X., Lu, Z., Meng, X., Wang, Y., Smith, S.M., and Li, J. (2015). Strigolactone signaling in Arabidopsis regulates shoot development by targeting D53-like SMXL repressor proteins for ubiquitination and degradation. *Plant Cell* 27**,** 3128-3142.

Xu, L., Liu, F., Lechner, E., Genschik, P., Crosby, W.L., Ma, H., Peng, W., Huang, D., and Xie, D. (2002). The SCF^COI1^ ubiquitin-ligase complexes are required for jasmonate response in Arabidopsis. *Plant Cell* 14**,** 1919-1935.

Yan, J., Li, X., Zeng, B., Zhong, M., Yang, J., Yang, P., Li, X., He, C., Lin, J., Liu, X., and Zhao, X. (2020). FKF1 F-box protein promotes flowering in part by negatively regulating DELLA protein stability under long-day photoperiod in Arabidopsis. *J Integr Plant Biol* 62**,** 1717-1740.

Ye, Q., Wang, H., Su, T., Wu, W.H., and Chen, Y.F. (2018). The ubiquitin E3 ligase PRU1 regulates WRKY6 degradation to modulate phosphate homeostasis in response to low-Pi stress in Arabidopsis. *Plant Cell* 30**,** 1062-1076.

Yu, S.-I., Kim, H., Yun, D.-J., Suh, M.C., and Lee, B.-H. (2019). Post-translational and transcriptional regulation of phenylpropanoid biosynthesis pathway by Kelch repeat F-box protein SAGL1. *Plant Mol Biol* 99**,** 135-148.

Zaltsman, A., Krichevsky, A., Loyter, A., and Citovsky, V. (2010). Agrobacterium induces expression of a host F-box protein required for tumorigenicity. *Cell Host Microbe* 7**,** 197-209.

Zhang, X., Abrahan, C., Colquhoun, T.A., and Liu, C.J. (2017). A proteolytic regulator controlling chalcone synthase stability and flavonoid biosynthesis in Arabidopsis. *Plant Cell* 29**,** 1157-1174.

Zhang, X., Gou, M., Guo, C., Yang, H., and Liu, C.J. (2015). Down-regulation of Kelch domain-containing F-box protein in Arabidopsis enhances the production of (poly)phenols and tolerance to ultraviolet radiation. *Plant Physiol* 167**,** 337-350.

Zhang, X., Gou, M., and Liu, C.J. (2013). Arabidopsis Kelch repeat F-box proteins regulate phenylpropanoid biosynthesis via controlling the turnover of phenylalanine ammonia-lyase. *Plant Cell* 25**,** 4994-5010.

Zhang, Y., Zhang, J., Guo, J., Zhou, F., Singh, S., Xu, X., Xie, Q., Yang, Z., and Huang, C.F. (2019). F-box protein RAE1 regulates the stability of the aluminum-resistance transcription factor STOP1 in Arabidopsis. *Proc Natl Acad Sci U S A* 116**,** 319-327.

Zhu, J.Y., Li, Y., Cao, D.M., Yang, H., Oh, E., Bi, Y., Zhu, S., and Wang, Z.Y. (2017). The F-box protein KIB1 mediates brassinosteroid-induced inactivation and degradation of GSK3-like kinases in Arabidopsis. *Mol Cell* 66**,** 648-657.e644.
