# Supplementary material for: A Machine Learning Approach to Prioritizing Functionally Active F-box Members in Arabidopsis thaliana
Source: Front Plant Sci. 2021 May 28;12:639253. doi: 10.3389/fpls.2021.639253 (PMC8192846; doi:10.3389/fpls.2021.639253)
Supplement: Supplementary file 6 [file Presentation_1.PDF]

```
#libraries

    library("tidyr")
    library("gplots")
    library("ConsensusClusterPlus")
    library("ggbiplot")
    library("neuralnet")


    # domain predictions for each FBX protein sequence
    dms<-read.table("Data_S2_FBX_domian_information.tab",header=T)

    # there are several FBXD HMM files which make slightly differnt predictions
    fbxds<-c("F-box","AO","AO_FBX","F-box-like")

    #get dm start, end, evalue
    dms<-dms %>% separate(DM, c("DM","evalue","range"), sep = "[|]")
    dms<-dms %>% separate(range, c("str","end"), sep = "[-]")

    #this study focuses on the FBX genes that have been annotated in TAIR and
predicted in our previous study
    #published in PLoS One (Hua et al., 2011) and IJMS (Hua, 2021).  14 were
removed due to missing of an FBXD upon reannotaion
    unique_fbxes<-unique(dms$PLoS_One_ID)
    length(unique_fbxes) #[1] 696

    dim(dms)[1] #[1] 2454 many FBXes have multiple CTDs
    length(table(dms$DM)) #[1] 238 total different CTDs


############# Publication data for each FBX gene and group information

    pub_fbxes<-read.table("Data_S1_Publication_number_comparison_in_four_groups_of_f
bx_genes.tab",header=T)

    pub_fbxes_plos<-pub_fbxes[pub_fbxes$PLoS_One_ID%in%unique_fbxes,]
    pub_fbxes_plos<-pub_fbxes_plos[order(pub_fbxes_plos$PLoS_One_ID),]

    group1<-pub_fbxes_plos[pub_fbxes_plos$group==1,]
    group2<-pub_fbxes_plos[pub_fbxes_plos$group==2,]
    group3<-pub_fbxes_plos[pub_fbxes_plos$group==3,]
    group4<-pub_fbxes_plos[pub_fbxes_plos$group==4,]

    group1_dms<-dms[substring(dms$AGI,1,9)%in%group1$AGI,]
    group2_dms<-dms[substring(dms$AGI,1,9)%in%group2$AGI,]
    group3_dms<-dms[substring(dms$AGI,1,9)%in%group3$AGI,]
    group4_dms<-dms[substring(dms$AGI,1,9)%in%group4$AGI,]
```

```
    unique_agi<-unique(pub_fbxes$AGI)
    length(unique_agi) #[1] 696


##########################################################################
#                                                                        #
# Figure 1  compare c-terminal domain frequency in four manually         #
#           defined groups according to prior studies                    #
#                                                                        #
##########################################################################

#function

    cdm_ratio<-function(dm){

            dms<-gsub("\\_.*","",dm$DM) #we combine all subdomains as one CTD
            dms<-data.frame(table(dms))
            dms<-dms[rev(order(dms$Freq)),]

            cdms<-dms[!dms$dms%in%fbxds,]

            rownames_cdms<-cdms$dms
            cdms<-as.matrix(cdms$Freq)
            rownames(cdms)<-rownames_cdms

            cdms_ratio<-cdms/colSums(cdms)

            cdms_ratio

        }

    # calculate the ratios of c-terminal domains in each group

    all_cdms<-cdm_ratio(dms)
    group1_cdms<-cdm_ratio(group1_dms)
    group2_cdms<-cdm_ratio(group2_dms)
    group3_cdms<-cdm_ratio(group3_dms)
    group4_cdms<-cdm_ratio(group4_dms)


    #compare top 10 c-terminal domains in each group
    all_cdms10<-all_cdms[1:10,]
    group1_cdms10<-group1_cdms[1:10,]
    group2_cdms10<-group2_cdms[1:10,]
    group3_cdms10<-group3_cdms[1:10,]
    group4_cdms10<-group4_cdms[1:10,]

    cdms10<-list(all_cdms10,group1_cdms10,group2_cdms10,group3_cdms10,group4_cdms10)
```

```
unique_cdms<-unique(c(names(all_cdms10),names(group1_cdms10),names(group2_cdms10
    ),names(group3_cdms10),names(group4_cdms10))) # in total 13 unique

#Make a data frame for the ratios of top 10 DMs in all and four separate groups

unique_cdms_df<-c()

for(i in 1:length(unique_cdms)){

    cdmi<-unique_cdms[i]

    cdmi_df<-c()

    for(j in 1:length(cdms10)){

        cdm10<-cdms10[[j]]
        cdm10_ratio<-cdm10[names(cdm10)%in%cdmi]
        if(length(cdm10_ratio) <1){cdm10_ratio<-0}
        cdmi_df<-cbind(cdmi_df,cdm10_ratio)

        }
    rownames(cdmi_df)<-cdmi
    unique_cdms_df<-rbind(unique_cdms_df,cdmi_df)

    }
colnames(unique_cdms_df)<-c("all","Group1","Group2","Group3","Group4")


#heatmap and hc clustering

d<-unique_cdms_df

rowDistance=dist(d,method="manhattan")
rowCluster = hclust(rowDistance,method="ward.D2")
rowDend = as.dendrogram(rowCluster)
rowDend = reorder(rowDend, rowSums(d))

colDistance=dist(t(d),method="manhattan")
colCluster = hclust(colDistance,method="ward.D2")
colDend = as.dendrogram(colCluster)
colDend = reorder(colDend, colSums(d))

my_palette<-colorRampPalette(c("light blue","yellow","red"))(n=19)
col_breaks=c(seq(0,0.001,length=5),seq(0.0011,0.25,length=10),seq(0.255,max(d),l
ength=5))

pdf ("Figures1B_CDM_comparison_heatmap.pdf", family="Times", height=5, width=5)
        par(mar=c(5.1,4.1,4.1,2.1), mgp=c(3,1,0), las=0)
        heatmap.2(d,Rowv=rowDend,
Colv=colDend,col=my_palette,breaks=col_breaks,key=FALSE,margins=c(3,3),trace=c("none
```

```
"),density.info=c("none"),cexRow=0.5,cexCol=0.5)
    dev.off()

    hc<-heatmap.2(d,Rowv=rowDend,
Colv=colDend,col=my_palette,breaks=col_breaks,key=FALSE,margins=c(3,3),trace=c("none
"),density.info=c("none"),cexRow=0.5,cexCol=0.5)
    t(hc$carpet)

                                     #
            #                        Group1     Group2      Group4         all
                Group3
            #          Arm        0.00000000 0.21621622 0.000000000 0.014532243
                0.000000000
            #          PP2        0.02298851 0.00000000 0.000000000 0.018165304
                0.023480663
            #          DUF295     0.02298851 0.02702703 0.000000000 0.037238874
                0.051104972
            #          Transp     0.08045977 0.00000000 0.000000000 0.006357856
                0.000000000
            #          WD40       0.00000000 0.04054054 0.000000000 0.005449591
                0.004143646
            #          Tub        0.00000000 0.04054054 0.000000000 0.009990917
                0.011049724
            #          PAS        0.03448276 0.00000000 0.000000000 0.000000000
                0.000000000
            #          TcpS       0.01149425 0.00000000 0.000000000 0.000000000
                0.000000000
            #          YcgR       0.01149425 0.00000000 0.000000000 0.000000000
                0.000000000
            #          Tn7        0.01149425 0.00000000 0.000000000 0.000000000
                0.000000000
            #          zf-ribbon 0.00000000 0.01351351 0.000000000 0.000000000
                0.000000000
            #          LBP        0.00000000 0.02702703 0.000000000 0.000000000
                0.000000000
            #          TetR       0.00000000 0.00000000 0.000000000 0.000000000
                0.004143646
            #          Sel1       0.00000000 0.00000000 0.000000000 0.000000000
                0.005524862
            #          DUF3794    0.00000000 0.00000000 0.009259259 0.000000000
                0.000000000
            #          SpoIIP     0.00000000 0.00000000 0.004629630 0.000000000
                0.000000000
            #          TLP-20     0.00000000 0.00000000 0.004629630 0.000000000
                0.000000000
            #          Toprim     0.00000000 0.00000000 0.004629630 0.000000000
                0.000000000
            #          Y1         0.00000000 0.00000000 0.004629630 0.000000000
                0.000000000
            #          Toxin      0.00000000 0.00000000 0.004629630 0.000000000
```

```
                0.000000000
            #           FBD       0.00000000 0.04054054 0.180555556 0.101725704
                0.096685083
            #           FBA       0.03448276 0.10810811 0.236111111 0.201634877
                0.220994475
            #           Kelch     0.40229885 0.06756757 0.074074074 0.207992734
                0.238950276
            #           LRR       0.28735632 0.28378378 0.250000000 0.188010899
                0.147790055


##########################################################################
#                                                                        #
#                   Machine Learning                                     #
#                                                                        #
##########################################################################
#functions

        #  We select the fbx genes predicted in >950 out of 1000 times of
           resampling
        #  as confident candidates

        predict<-function(predicted){

            predicted_cnt<-as.matrix(table(predicted))
            predicted_cnt<-as.matrix (predicted_cnt[rev( order(predicted_cnt[,1])
), ])

            list<-seq(from=1,to=dim(predicted_cnt)[1],by=1)
            predicted_cnt<-cbind(list,predicted_cnt)
            constant_predicted<-predicted_cnt[predicted_cnt[,2]>950,]

            constant_predicted

            }

        # Distribution of four groups of FBXes in Km clusters

        group_in_clusters_fun<-function(clusters){

            group_in_clusters<-c()

            for(i in 1:4){

                cluster_i<-clusters[clusters[,1]==i,]

                g1<-cluster_i[rownames(cluster_i)%in%group1$PLoS_One_ID,]
                g2<-cluster_i[rownames(cluster_i)%in%group2$PLoS_One_ID,]
                g3<-cluster_i[rownames(cluster_i)%in%group3$PLoS_One_ID,]
                g4<-cluster_i[rownames(cluster_i)%in%group4$PLoS_One_ID,]
```

```
                cnt1<-dim(g1)[1]
                cnt2<-dim(g2)[1]
                cnt3<-dim(g3)[1]
                cnt4<-dim(g4)[1]

                if(is.null(cnt1)){cnt1<-0}
                if(is.null(cnt2)){cnt2<-0}
                if(is.null(cnt3)){cnt3<-0}
                if(is.null(cnt4)){cnt4<-0}


                group_in_cluster_i<-cbind(round(cnt1/dim(group1)[1],2),
                                          round(cnt2/dim(group2)[1],2),
                                          round(cnt3/dim(group3)[1],2),
                                          round(cnt4/dim(group4)[1],2)
                                                                  )

                group_in_clusters<-rbind(group_in_clusters,group_in_cluster_i)

                }

                rownames(group_in_clusters)<-c("cluster1","cluster2","cluster3","clu
ster4")
                colnames(group_in_clusters)<-c("group1","group2","group3","group4")

                group_in_clusters

            }



####################################

# Method 1, everyting is included

        fbx_seq_infor_df<-read.table("Data_S3_fbx_multi_dimensional_features.tab",he
ader=T)

        fbx_seq_infor_wo_agi_m1_df<-fbx_seq_infor_df[,-1]

        #order the FBXes based on group number
        fbx_seq_infor_wo_agi_m1_df<-fbx_seq_infor_wo_agi_m1_df[order(fbx_seq_infor_w
o_agi_m1_df$group),]

        colnames(fbx_seq_infor_wo_agi_m1_df)

                        #
                #       [1] "group"                     "Publications"          "EST"

                #        [4] "cDNA"                      "Intron"
```

```
                     "Total_TDNA"
                 #        [7] "TDNA_100upstream"       "TDNA_First_half_intron"
                     "TDNA_First_half_exon"
                 #       [10] "TDNA_Last_half_intron"  "TDNA_Last_half_exon"
                    "kaks"
                 #       [13] "ks"                     "neutral"
                    "cdms_rate"
                 #       [16] "fbxd_evalue"            "fbxd_str"
                    "fbxd_end"
                 #       [19] "length"                 "Kelch"
                    "FBA"
                 #       [22] "LRR"                    "FBD"
                    "fbx_exp_mean"
                 #       [25] "fbx_exp_median"         "fbx_exp_max"
                    "fbx_exp_cv"


######### unsupervised K-means clustering

        d<-scale(fbx_seq_infor_wo_agi_m1_df)
        d<-d[,-1]
        d<-t(d)

        title<-getwd()

        #resampling k-means clustering
        results =
ConsensusClusterPlus(d,maxK=9,reps=1000,pItem=0.8,pFeature=1,title=title,
                                        innerLinkage="average",finalLinkage="average
",clusterAlg="km",
                                        distance="euclidean",seed=1262118388.71279,p
lot="png")

        clusters<-as.matrix(results[[4]][["consensusClass"]])

        clusters<-cbind(clusters,paste("c",clusters[,1],sep="_"))

        clusters_27features<-clusters

        group_in_clusters<-group_in_clusters_fun(clusters_27features)
        group_in_clusters

                                        #
                     #               group1 group2 group3 group4
                     #      cluster1   0.78   0.59   0.15   0.00
                     #      cluster2   0.05   0.05   0.23   0.24
                     #      cluster3   0.15   0.29   0.47   0.46
                     #      cluster4   0.00   0.07   0.16   0.29
```

```
            pdf("Figure
3A_fbx_seq_infor_wo_agi_df_27features_scale_km_barplot.pdf",width=5,height=5)

                    barplot(t(group_in_clusters),
col=c("gray","red","cyan","brown"), main="method1_km_clusters",
                              xlab="Clusters",ylim=c(0,1), beside=TRUE,cex.axis=0.3,
cex.names=0.4,legend = colnames(group_in_clusters))
            dev.off()
    ##########


        #library("ggbiplot")

        fbx_seq_infor_wo_agi_m1_df.pca <- prcomp(fbx_seq_infor_wo_agi_m1_df, center
= TRUE, scale. = TRUE)

        summary(fbx_seq_infor_wo_agi_m1_df.pca)

                                        #
                                #       Importance of components:
                                #                                 PC1     PC2     PC3
                                      PC4     PC5     PC6     PC7
                                #       Standard deviation     2.3229 1.61295 1.45526
                                   1.40020 1.22501 1.18764 1.09905
                                #       Proportion of Variance 0.1998 0.09636 0.07844
                                   0.07261 0.05558 0.05224 0.04474
                                #       Cumulative Proportion  0.1998 0.29621 0.37465
                                   0.44726 0.50284 0.55508 0.59981
                                #                                  PC8     PC9    PC10
                                     PC11    PC12    PC13    PC14
                                #       Standard deviation     1.06186 1.03591 0.9968
                                   0.97238 0.91654 0.88359 0.83676
                                #       Proportion of Variance 0.04176 0.03974 0.0368
                                   0.03502 0.03111 0.02892 0.02593
                                #       Cumulative Proportion  0.64158 0.68132 0.7181
                                   0.75314 0.78425 0.81317 0.83910
                                #                                 PC15    PC16    PC17
                                     PC18    PC19    PC20    PC21
                                #       Standard deviation     0.77295 0.75136 0.7403
                                   0.70615 0.68792 0.65002 0.60682
                                #       Proportion of Variance 0.02213 0.02091 0.0203
                                   0.01847 0.01753 0.01565 0.01364
                                #       Cumulative Proportion  0.86123 0.88214 0.9024
                                   0.92091 0.93843 0.95408 0.96772
                                #                                 PC22   PC23    PC24
                                     PC25    PC26      PC27
                                #       Standard deviation     0.55250 0.5376 0.51366
                                   0.08881 0.07457 3.207e-16
                                #       Proportion of Variance 0.01131 0.0107 0.00977
```

```
                                0.00029 0.00021 0.000e+00
                             #       Cumulative Proportion  0.97903 0.9897 0.99950
                                0.99979 1.00000 1.000e+00


        pdf("Figure
3B_fbx_seq_infor_wo_agi_df_27features_scale_km.pca.pdf",width=5,height=5)
            ggbiplot(fbx_seq_infor_wo_agi_m1_df.pca,ellipse=TRUE,obs.scale = 2,
var.scale = 1, var.axes=FALSE,
                labels=fbx_seq_infor_wo_agi_m1_df[,1],groups= clusters[,2]) +
            scale_colour_manual(name="Origin", values= c("purple", "forest green",
"blue","magenta"))+
            ggtitle("PCA of ortho_spec dataset")+
            theme_classic()+
            theme(legend.position = "bottom")
        dev.off()




########## Neural network

        scaleddata<-scale(fbx_seq_infor_wo_agi_m1_df)
        colnames(scaleddata)

        x<-scaleddata[1:82,]
        y<-scaleddata[83:692,]

        x_group<-rep(1,dim(x)[1]) #taking Groups 1 and 2 as known FBXes, labelled
as 1
        y_group<-rep(0,dim(y)[1]) #taking Groups 3 and 4 as unknown FBXes, labelled
as 0

        x<-cbind(x_group,x[,-1])
        y<-cbind(y_group,y[,-1])

        colnames(x)<-gsub("x_group","activity",colnames(x))

        colnames(y)<-gsub("y_group","activity",colnames(y))

        x<-data.frame(x)
        y<-data.frame(y)

        x1<-x[1:41,]
        x2<-x[42:dim(x)[1],]
        y1<-y[1:470,]
        y2<-y[471:dim(y)[1],]
```

```
    ####################################################################################
        ################

        #training and neural decision tree

        ###### sample and split data

        #we treat group 4 FBXes as non functional (marked as 0) due to the lack of
any studies
        #We consider group 1 FBXes as active FBX proteins (marked as 1) since both
biochemical and functional studies have been done.
        #Since there are 41 group 1 FBXes, we took 3 x 41 = 123 group 4 FBXes to
combine with group 1 as training and validating datasets

        sample <-sample(nrow(y2),size=123,replace=F,)
        y2_tr_va <- y2[sample, ] # train_validate

        #we treat groups 2 and 3 FBX genes as unknown samples for testing
        testdata <-rbind(x2,y1)

        tr_va <- rbind(x1,y2_tr_va)       ###########  (tr)an+(va)lide dataset

        #we take train/validate=2:1 ratio (41+123=164)
        number <- sample(nrow(tr_va), size =109, replace = F, )
        train <- tr_va[number,]  #109 train
        validate <- tr_va[-number,]  #55 validate


        rand<-sample(seq(from=2000,to=30000,by=1),1,replace=F)
        set.seed(rand)

        nn <- neuralnet(activity~., data=train, hidden=c(10,2),
linear.output=FALSE, threshold=0.01)

        par(mar = c(10, 5, 5, 5)) # Set the margin on all sides to 6
        plot(nn)



        ##############
        # "positive" means "functionally active"
        # "negative" means "functionally inactive"

        constant_positive_prediction<-c()
        constant_negative_prediction<-c()
        constant_top_ten_percent<-c()
        constant_bottom_ten_percent<-c()

        mean_accuracy<-c()
        mean_true_prediction<-c()
```

```
        mean_false_n_p_rates<-c()


        for(j in 1:10){

                accuracy<-c()
                positive<-c()
                negative<-c()
                true_prediction<-c()
                false_n_p_rates<-c()

                for (i in 1:1000){


                    sample <-sample(nrow(y2),size=123,replace=F,)
                    y2_tr_va <- y2[sample, ] # validate

                    #we treat groups 2 and 3 FBX genes as unknown for testing
                    testdata <-rbind(x2,y1)

                    tr_va <- rbind(x1,y2_tr_va)       ###########  (tr)an+(va)lide
                        dataset 41 +123=164

                    #we take train/validate=2:1 ratio
                    number <- sample(nrow(tr_va), size =109, replace = F, )
                    train <- tr_va[number,] #109
                    validate <- tr_va[-number,]  #55


                    rand<-sample(seq(from=2000,to=30000,by=1),1,replace=F)
                    set.seed(rand)

                    validate_x1<-validate[rownames(validate)%in%rownames(x1),]
                    validate_y2<-validate[rownames(validate)%in%rownames(y2),]

                    nn <- neuralnet(activity~., data=train, hidden=c(10,2),
linear.output=FALSE, threshold=0.01)

                    val_output <- compute(nn, validate[, -1])
                    val_pred <- val_output$net.result
                    val_pred <- ifelse(val_pred>0.5, 1, 0)
                    tab <- table(val_pred, validate[,1])
                    accu<-1-sum(diag(tab))/sum(tab)
                    accuracy<-rbind(accuracy,accu)

                    false_n_p<-cbind(tab[1,2]/dim(validate_x1)[1],tab[2,1]/dim(valid
ate_y2)[1])
                    false_n_p_rates<-rbind(false_n_p_rates,false_n_p)

                     #### final test ####
```

```
                    # test
                    test <- compute(nn, testdata[, -1])
                    test_prediction <- test$net.result
                    test_prediction_adj <- ifelse(test_prediction>0.5, 1, 0)

                    rownames(test_prediction_adj)<-rownames(test_prediction)
                    true_positive<-names(test_prediction_adj[test_prediction_adj[,1]
>0,])
                    true_negative<-names(test_prediction_adj[test_prediction_adj[,1]
<1,])

                    positive<-rbind(positive,as.matrix(true_positive))
                    negative<-rbind(negative,as.matrix(true_negative))


                    #how often are FBX genes discovered as active members at one run
                    true_x2<-length(true_positive[true_positive%in%rownames(x2)])
                    true_x2_rate<-true_x2/dim(x2)[1]

                    true_y1<-length(true_positive[true_positive%in%rownames(y1)])
                    true_y1_rate<-true_y1/dim(y1)[1]


                    true_prediction_rate<-cbind(true_x2_rate,true_y1_rate)

                    true_prediction<-rbind(true_prediction,true_prediction_rate)


                    }

            mean_accuracy<-rbind(mean_accuracy,mean(accuracy))

            mean_true_prediction<-rbind(mean_true_prediction,colMeans(true_predictio
n))
            mean_false_n_p_rates<-rbind(mean_false_n_p_rates,colMeans(false_n_p_rate
s))

            constant_positive<-predict(positive)
            constant_negative<-predict(negative)

            constant_positive_prediction<-rbind(constant_positive_prediction,constan
t_positive)

            constant_negative_prediction<-rbind(constant_negative_prediction,constan
t_negative)

        }
```

```
        m1_mean_accuracy<-(1-mean_accuracy[,1])*100
        m1_prediction_rates<-mean_true_prediction
        m1_false_n_p_rates<-mean_false_n_p_rates


        m1_positive_predictions<-as.matrix(table(rownames(constant_positive_predicti
on)))
        m1_positive_predictions<-m1_positive_predictions[m1_positive_predictions[,1]
>9,]

        length(m1_positive_predictions) #52

        positive_names<-names(m1_positive_predictions)
        group1_names<-rownames(group1)

        write.table(as.matrix(c(group1_names,positive_names)),"m1_positive_predictio
ns_for_phylogenetic_analysis.tab",sep="\t")


        m1_negative_predictions<-as.matrix(table(rownames(constant_negative_predicti
on)))
        m1_negative_predictions<-m1_negative_predictions[m1_negative_predictions[,1]
>9,]

        length(m1_negative_predictions) #[1] 210
        m1_negative_names<-names(m1_negative_predictions)


        #compair with Km clustering
        m1_positive_predictions_in_km_clusters<-clusters_27features[rownames(cluster
s_27features)%in%names(m1_positive_predictions),]
        table(m1_positive_predictions_in_km_clusters[,2])/sum(table(m1_positive_pred
ictions_in_km_clusters[,2]))

                       #
            #        c_1       c_3
            #  0.8846154 0.1153846



        m1_negative_predictions_in_km_clusters<-clusters_27features[rownames(cluster
s_27features)%in%names(m1_negative_predictions),]
        table(m1_negative_predictions_in_km_clusters[,2])/sum(table(m1_negative_pred
ictions_in_km_clusters[,2]))
                       #
            #        c_2       c_3       c_4
            #  0.2619048 0.4523810 0.2857143
```

```
#####################

# Method 2, remove ambiguous features based on learning

        fbx_seq_infor_wo_agi_m2_df<-fbx_seq_infor_wo_agi_m1_df[,-c(6:11,14:23)]
        colnames(fbx_seq_infor_wo_agi_m2_df)

             #   [1] "group"          "Publications"   "EST"             "cDNA"

             #   [5] "Intron"         "kaks"           "ks"
                 "fbx_exp_mean"
             #   [9] "fbx_exp_median" "fbx_exp_max"    "fbx_exp_cv"

######### unsupervised K-means clustering

        d<-scale(fbx_seq_infor_wo_agi_m2_df)
        d<-d[,-1]
        d<-t(d)

        title<-getwd()

        #resampling k-means clustering
        results =
ConsensusClusterPlus(d,maxK=9,reps=1000,pItem=0.8,pFeature=1,title=title,
                                        innerLinkage="average",finalLinkage="average
",clusterAlg="km",
                                        distance="euclidean",seed=1262118388.71279,p
lot="png")

        clusters<-as.matrix(results[[4]][["consensusClass"]])
        clusters<-cbind(clusters,paste("c",clusters[,1],sep="_"))

        clusters_10features<-clusters

        group_in_clusters<-group_in_clusters_fun(clusters_10features)
        group_in_clusters

                    #               group1 group2 group3 group4
                    #      cluster1   0.44   0.22   0.04   0.00
                    #      cluster2   0.44   0.49   0.26   0.09
                    #      cluster3   0.12   0.29   0.70   0.91
                    #      cluster4   0.00   0.00   0.00
                        0.00                                  #


        pdf("Figure
3C_fbx_seq_infor_wo_agi_df_10features_scale_km_barplot.pdf",width=5,height=5)

            barplot(t(group_in_clusters), col=c("gray","red","cyan","brown"),
main="method2_km",
```

```
                 xlab="Clusters",ylim=c(0,1), beside=TRUE,cex.axis=0.3,
cex.names=0.4,)

        dev.off()

###########

        fbx_seq_infor_wo_agi_m2_df.pca <- prcomp(fbx_seq_infor_wo_agi_m2_df[,-1],
center = TRUE, scale. = TRUE)

        summary(fbx_seq_infor_wo_agi_m2_df.pca)

                                #
                        #       Importance of components:
                        #                              PC1    PC2    PC3    PC4
                             PC5     PC6     PC7
                        #       Standard deviation     2.074 1.0811 1.0345 0.9731
                           0.92903 0.76446 0.63470
                        #       Proportion of Variance 0.430 0.1169 0.1070 0.0947
                           0.08631 0.05844 0.04028
                        #       Cumulative Proportion  0.430 0.5469 0.6539 0.7486
                           0.83490 0.89334 0.93363
                        #                                PC8     PC9    PC10
                        #       Standard deviation     0.59925 0.54652 0.07703
                        #       Proportion of Variance 0.03591 0.02987 0.00059
                        #       Cumulative Proportion  0.96954 0.99941 1.00000




        pdf("Figure
3D_fbx_seq_infor_wo_agi_m2_df_10features_scale.pca2.pdf",width=5,height=5)
            ggbiplot(fbx_seq_infor_wo_agi_m2_df.pca,ellipse=TRUE,obs.scale = 2,
var.scale = 1, var.axes=TRUE,
                labels=fbx_seq_infor_wo_agi_m2_df[,1],groups= clusters[,2]) +
            scale_colour_manual(name="Origin", values= c("purple", "forest green",
"blue","magenta"))+
            ggtitle("PCA of ortho_spec dataset")+
            theme_classic()+
            theme(legend.position = "bottom")

        dev.off()



#####################


        scaleddata<-scale(fbx_seq_infor_wo_agi_m2_df)
        colnames(scaleddata)
```

```
        #
    #    [1] "group"         "Publications"   "EST"             "cDNA"

    #    [5] "Intron"        "kaks"           "ks"
        "fbx_exp_mean"
    #    [9] "fbx_exp_median" "fbx_exp_max"    "fbx_exp_cv"

x<-scaleddata[1:82,]
y<-scaleddata[83:692,]

x_group<-rep(1,dim(x)[1])
y_group<-rep(0,dim(y)[1])

x<-cbind(x_group,x[,-1])
y<-cbind(y_group,y[,-1])

colnames(x)<-gsub("x_group","activity",colnames(x))

colnames(y)<-gsub("y_group","activity",colnames(y))

x<-data.frame(x)
y<-data.frame(y)

x1<-x[1:41,]
x2<-x[42:dim(x)[1],]
y1<-y[1:470,]
y2<-y[471:dim(y)[1],]
############################################################################
    ####################

#training and neural decision tree

###### sample and splite data

#we treat group 4 FBXes as non functional (marked as 0) due to the lack of
any studies
#We consider group 1 FBXes as active FBX proteins (marked as 1) since both
biochemical and functional studies have been done
#Since there are 41 group 1 FBXes, we took 3 x 41 = 123 group 4 FBXes to
combine with group 1 as training and validating datasets

sample <-sample(nrow(y2),size=123,replace=F,)
y2_tr_va <- y2[sample, ] # validate

#we treat groups 2 and 3 FBX genes as unknown for testing
testdata <-rbind(x2,y1)

tr_va <- rbind(x1,y2_tr_va)      ###########  (tr)an+(va)lide dataset

#we take train/validate=2:1 ratio
```

```
        number <- sample(nrow(tr_va), size =109, replace = F, )
        train <- tr_va[number,]
        validate <- tr_va[-number,]


        rand<-sample(seq(from=2000,to=30000,by=1),1,replace=F)
        set.seed(rand)

        nn <- neuralnet(activity~., data=train, hidden=c(10,2),
linear.output=FALSE, threshold=0.01)

        par(mar = c(5, 5, 5, 5)) # Set the margin on all sides to 6
        plot(nn)



############## neural network predictions
        # "positive" means "functionally active"
        # "negative" means "functionally inactive"

        constant_positive_prediction<-c()
        constant_negative_prediction<-c()
        constant_top_ten_percent<-c()
        constant_bottom_ten_percent<-c()

        mean_accuracy<-c()
        mean_true_prediction<-c()
        mean_false_n_p_rates<-c()


        for(j in 1:10){

                accuracy<-c()
                positive<-c()
                negative<-c()
                true_prediction<-c()
                false_n_p_rates<-c()

                for (i in 1:1000){


                    sample <-sample(nrow(y2),size=123,replace=F,)
                    y2_tr_va <- y2[sample, ] # validate

                    #we treat groups 2 and 3 FBX genes as unknown for testing
                    testdata <-rbind(x2,y1)

                    tr_va <- rbind(x1,y2_tr_va)       ###########  (tr)an+(va)lide
                        dataset
```

```
#we take train/validate=2:1 ratio
number <- sample(nrow(tr_va), size =109, replace = F, )
train <- tr_va[number,]
validate <- tr_va[-number,]


rand<-sample(seq(from=2000,to=30000,by=1),1,replace=F)
set.seed(rand)

validate_x1<-validate[rownames(validate)%in%rownames(x1),]
validate_y2<-validate[rownames(validate)%in%rownames(y2),]

nn <- neuralnet(activity~., data=train, hidden=c(10,2),
linear.output=FALSE, threshold=0.01)

val_output <- compute(nn, validate[, -1])
val_pred <- val_output$net.result
val_pred <- ifelse(val_pred>0.5, 1, 0)
tab <- table(val_pred, validate[,1])
accu<-1-sum(diag(tab))/sum(tab)
accuracy<-rbind(accuracy,accu)

false_n_p<-cbind(tab[1,2]/dim(validate_x1)[1],tab[2,1]/dim(valid
ate_y2)[1])

false_n_p_rates<-rbind(false_n_p_rates,false_n_p)

 #### final test ####


# test
test <- compute(nn, testdata[, -1])
test_prediction <- test$net.result
test_prediction_adj <- ifelse(test_prediction>0.5, 1, 0)

rownames(test_prediction_adj)<-rownames(test_prediction)
true_positive<-names(test_prediction_adj[test_prediction_adj[,1]
>0,])

true_negative<-names(test_prediction_adj[test_prediction_adj[,1]
<1,])


positive<-rbind(positive,as.matrix(true_positive))
negative<-rbind(negative,as.matrix(true_negative))


#how often are FBX genes discovered as active members at one run
true_x2<-length(true_positive[true_positive%in%rownames(x2)])
true_x2_rate<-true_x2/dim(x2)[1]

true_y1<-length(true_positive[true_positive%in%rownames(y1)])
true_y1_rate<-true_y1/dim(y1)[1]
```

```
                true_prediction_rate<-cbind(true_x2_rate,true_y1_rate)

                true_prediction<-rbind(true_prediction,true_prediction_rate)


                }

            mean_accuracy<-rbind(mean_accuracy,mean(accuracy))

            mean_true_prediction<-rbind(mean_true_prediction,colMeans(true_predictio
n))
            mean_false_n_p_rates<-rbind(mean_false_n_p_rates,colMeans(false_n_p_rate
s))

            constant_positive<-predict(positive)
            constant_negative<-predict(negative)

            constant_positive_prediction<-rbind(constant_positive_prediction,constan
t_positive)

            constant_negative_prediction<-rbind(constant_negative_prediction,constan
t_negative)


        }



        m2_positive_predictions<-as.matrix(table(rownames(constant_positive_predicti
on)))
        m2_positive_predictions<-m2_positive_predictions[m2_positive_predictions[,1]
>9,]

        m1_m2_positive_predictions<-m1_positive_predictions[names(m1_positive_predic
tions)%in%names(m2_positive_predictions)]

        length(m1_m2_positive_predictions) #37
        length(m2_positive_predictions) #54


        #Compare with Km clustering
        m2_positive_predictions_in_km_clusters<-clusters_10features[rownames(cluster
s_10features)%in%names(m2_positive_predictions),]
        table(m2_positive_predictions_in_km_clusters[,2])/sum(table(m2_positive_pred
ictions_in_km_clusters[,2]))

                #
            #         c_1        c_2
```

```
            #        0.4074074 0.5925926


        positive_names<-names(m2_positive_predictions)
        group1_names<-rownames(group1)

        write.table(as.matrix(c(group1_names,positive_names)),"m2_positive_predictio
ns_for_phylogenetic_analysis.tab",sep="\t")


        m2_negative_predictions<-as.matrix(table(rownames(constant_negative_predicti
on)))
        m2_negative_predictions<-m2_negative_predictions[m2_negative_predictions[,1]
>9,]

        length(m2_negative_predictions) #[1] 247

        m2_negative_predictions_in_km_clusters<-clusters_10features[rownames(cluster
s_10features)%in%names(m2_negative_predictions),]
        table(m2_negative_predictions_in_km_clusters[,2])/sum(table(m2_negative_pred
ictions_in_km_clusters[,2]))

                    #
            #        c_2         c_3
            # 0.09716599 0.90283401



        m1_m2_negative_predictions<-m1_negative_predictions[names(m1_negative_predic
tions)%in%names(m2_negative_predictions)]

        length(m1_m2_negative_predictions) #160


        negative_names<-names(m2_negative_predictions)

        rand_m2_negative<-sample(negative_names,size=length(m2_positive_predictions)
,replace=F)
        write.table(as.matrix(c(rownames(group1),rand_m2_negative)),"m2_negative_pre
dictions_for_phylogenetic_analysis.tab",sep="\t")





        m2_mean_accuracy<-(1-mean_accuracy[,1])*100

        m2_prediction_rates<-mean_true_prediction

        m2_false_n_p_rates<-mean_false_n_p_rates
```

```r
########Comparison between Method 1 and Method 2

        library("ggpubr")

#1) Prediction accuracy
        m1_accu_df<-data.frame(Measure=m1_mean_accuracy,Group="Method 1")
        m2_accu_df<-data.frame(Measure=m2_mean_accuracy,Group="Method 2")

        m1_m2_mean_accuracy_df<-rbind(m1_accu_df,m2_accu_df)

        attach(m1_m2_mean_accuracy_df)



        pdf("Figure4A_Prediction accuracy.pdf",height=5,width=5)

        ggboxplot(m1_m2_mean_accuracy_df, x = "Group", y = "Measure",
                  color = "Group", palette = c("gray","#6a0dad" ),
                  order = c("Method 1","Method 2"),
                  ylab = "Accuracy (%)", xlab = "Method",ylim=c(85,100))

        dev.off()

        t.test(m1_mean_accuracy, m2_mean_accuracy, alternative = c("less"), mu = 0,
               paired = FALSE, var.equal = FALSE, conf.level = 0.95)

                        #
            #           Welch Two Sample t-test

            #       data:  m1_mean_accuracy and m2_mean_accuracy
            #       t = -387.56, df = 12.743, p-value < 2.2e-16
            #       alternative hypothesis: true difference in means is less than 0
            #       95 percent confidence interval:
            #             -Inf -5.676991
            #       sample estimates:
            #       mean of x mean of y
            #        90.23345  95.93655




# 2) False predictions

        m1_false_n<-m1_false_n_p_rates[,1]*100
        m1_false_p<-m1_false_n_p_rates[,2]*100
        m2_false_n<-m2_false_n_p_rates[,1]*100
        m2_false_p<-m2_false_n_p_rates[,2]*100
```

```
        m1_false_n_df<-data.frame(Measure=m1_false_n,Group="m1_false_n")
        m1_false_p_df<-data.frame(Measure=m1_false_p,Group="m1_false_p")
        m2_false_n_df<-data.frame(Measure=m2_false_n,Group="m2_false_n")
        m2_false_p_df<-data.frame(Measure=m2_false_p,Group="m2_false_p")


        m1_m2_false_p_n_df<-rbind(m1_false_n_df,m2_false_n_df,m1_false_p_df,m2_false
_p_df)

        attach(m1_m2_false_p_n_df)


        pdf("Figure4B_False negative and positive prediction
rates.pdf",height=5,width=5)
                ggboxplot(m1_m2_false_p_n_df, x = "Group", y = "Measure",
                          color = "Group", palette =
c("gray","#6a0dad","gray","#6a0dad"),
                          order =
c("m1_false_n","m2_false_n","m1_false_p","m2_false_p"),
                          ylab = "False Prediction Rate (%)", xlab =
"Method",ylim=c(0,25))
        dev.off()

        #False negative

        t.test(m1_false_n, m2_false_n, alternative = c("greater"), mu = 0,
                       paired = FALSE, var.equal = FALSE, conf.level = 0.95)

                                #
                    #           Welch Two Sample t-test

                    #       data:  m1_false_n and m2_false_n
                    #       t = 180.04, df = 10.298, p-value < 2.2e-16
                    #       alternative hypothesis: true difference in means is
                       greater than 0
                    #       95 percent confidence interval:
                    #        8.73405     Inf
                    #       sample estimates:
                    #       mean of x mean of y
                    #        20.60667  11.78407

        #False positive
        t.test(m1_false_p, m2_false_p, alternative = c("greater"), mu = 0,
                   paired = FALSE, var.equal = FALSE, conf.level = 0.95)

                            #
                    #       Welch Two Sample t-test
```

```
                    #   data:  m1_false_p and m2_false_p
                    #   t = 311.5, df = 11.106, p-value < 2.2e-16
                    #   alternative hypothesis: true difference in means is greater
                      than 0
                    #   95 percent confidence interval:
                    #    4.946522      Inf
                    #   sample estimates:
                    #   mean of x mean of y
                    #    6.258016  1.282836


#3) Prediction rates

        m1_x2_prediction<-m1_prediction_rates[,1]*100
        m1_y1_prediction<-m1_prediction_rates[,2]*100
        m2_x2_prediction<-m2_prediction_rates[,1]*100
        m2_y1_prediction<-m2_prediction_rates[,2]*100


        m1_x2_predictino_df<-data.frame(Measure=m1_x2_prediction,Group="m1_x2")
        m1_y1_predictino_df<-data.frame(Measure=m1_y1_prediction,Group="m1_y1")
        m2_x2_prediction_df<-data.frame(Measure=m2_x2_prediction,Group="m2_x2")
        m2_y1_prediction_df<-data.frame(Measure=m2_y1_prediction,Group="m2_y1")


        m1_m2_prediction_df<-rbind(m1_x2_predictino_df,m2_x2_prediction_df,m1_y1_pre
dictino_df,m2_y1_prediction_df)

        attach(m1_m2_prediction_df)


        pdf("Figure4C_Top prediction rates_x2_y1_groups.pdf",height=5,width=5)

            ggboxplot(m1_m2_prediction_df, x = "Group", y = "Measure",
                  color = "Group", palette = c("gray","#6a0dad","gray","#6a0dad"),
                  order = c("m1_x2","m2_x2","m1_y1","m2_y1"),
                  ylab = "Prediction Rate (%)", xlab = "Method",ylim=c(0,80))
        dev.off()

        #Group 2 prediction rate (%)

        t.test(m1_x2_prediction, m2_x2_prediction, alternative = c("greater"), mu =
0,
                         paired = FALSE, var.equal = FALSE, conf.level = 0.95)

                                  #
                    #             Welch Two Sample t-test
                    #
                    #       data:  m1_x2_prediction and m2_x2_prediction
```

```
#       t = -7.2823, df = 9.9553, p-value = 1
#       alternative hypothesis: true difference in means is
   greater than 0
#       95 percent confidence interval:
#        -0.6205393        Inf
#       sample estimates:
#       mean of x mean of y
#        61.80415  62.30098


#Group 3 prediction rate (%)

 t.test(m1_y1_prediction, m2_y1_prediction, alternative = c("greater"), mu
= 0,
          paired = FALSE, var.equal = FALSE, conf.level = 0.95)

        #
#       Welch Two Sample t-test

#   data:  m1_y1_prediction and m2_y1_prediction
#   t = 38.402, df = 11.776, p-value = 4.852e-14
#   alternative hypothesis: true difference in means is greater
  than 0
#   95 percent confidence interval:
#    1.059457      Inf
#   sample estimates:
#   mean of x mean of y
#    25.72749  24.61638


#Final prediction list


x2_positive_predictions<-m2_positive_predictions[names(m2_positive_predictio
ns)%in%rownames(x2)]
y1_positive_predictions<-m2_positive_predictions[names(m2_positive_predictio
ns)%in%rownames(y1)]

length(x2_positive_predictions) #15
length(y1_positive_predictions) #39



###########

x2_negative_predictions<-m2_negative_predictions[names(m2_negative_predictio
ns)%in%rownames(x2)]
y1_negative_predictions<-m2_negative_predictions[names(m2_negative_predictio
ns)%in%rownames(y1)]
```

```
	length(x2_negative_predictions) #4
	length(y1_negative_predictions) #243


	########### Write out the prediction result
	x2_positive_group<-rep("Group 
II_Active",length(x2_positive_predictions))
	x2_positive<-cbind(names(x2_positive_predictions),x2_positive_group)

	x2_negative_group<-rep("Group II_Inactive",length(x2_negative_predictions))
	x2_negative<-cbind(names(x2_negative_predictions),x2_negative_group)

	y1_positive_group<-rep("Group 
III_Active",length(y1_positive_predictions))
	y1_positive<-cbind(names(y1_positive_predictions),y1_positive_group)

	y1_negative_group<-rep("Group III_Inactive",length(y1_negative_predictions))
	y1_negative<-cbind(names(y1_negative_predictions),y1_negative_group)


	prediction<-rbind(x2_positive,x2_negative,y1_positive,y1_negative)

	colnames(prediction)<-c("FBX_ID","Activity_Prediction")

	write.csv(prediction,"Data_S4_ANN_predictions_w_10_characteristics.csv")
```
