# Supplementary material for: A Machine Learning Approach to Prioritizing Functionally Active F-box Members in Arabidopsis thaliana
Source: Front Plant Sci. 2021 May 28;12:639253. doi: 10.3389/fpls.2021.639253 (PMC8192846; doi:10.3389/fpls.2021.639253)
Supplement: Supplementary file 7 [file Presentation_2.PDF]

```r
#functions

    predict_stat<-function(predicted){

        predicted_cnt<-as.matrix(table(predicted))
        predicted_cnt<-as.matrix (predicted_cnt[rev( order(predicted_cnt[,1]) ), ])

        list<-seq(from=1,to=dim(predicted_cnt)[1],by=1)
        predicted_cnt<-cbind(list,predicted_cnt)
        constant_predicted<-predicted_cnt[predicted_cnt[,2]>950,]

        constant_predicted

        }

# Machine learning 1: SVM

        #read in data

        data0 <- read.table("Data_S3_fbx_multi_dimensional_features.tab",header= T)
        data0 <-data0[,-1]

        data0<-data0[,-c(6:11,14:23)]
        colnames(data0)

                #    [1] "group"          "Publications"   "EST"              "cDNA"

            #    [5] "Intron"         "kaks"           "ks"
                "fbx_exp_mean"
            #    [9] "fbx_exp_median" "fbx_exp_max"    "fbx_exp_cv"

        #library
        library("e1071")

        #group data
        x1 <- data0[data0$group==1,]
        x2 <- data0[data0$group==2,]
        y1 <- data0[data0$group==3,]
        y2 <- data0[data0$group==4,]


        #prediction
        constant_positive_prediction<-c()
        constant_negative_prediction<-c()

        mean_accuracy<-c()
        mean_true_prediction<-c()
        mean_false_n_p_rates<-c()
```

```
    for(j in 1:10){  #10 rounds

        accuracy<-c()
        positive<-c()
        negative<-c()
        true_prediction<-c()
        false_n_p_rates<-c()


        for (i in 1:1000){  #1000 resampling

            sample <-sample(nrow(y2),size=123,replace=F,)
            y2_tr_va <- y2[sample, ] # validate
            y2_test<-y2[-sample,]

            #we treat groups 2 and 3 FBX genes as unknown for testing
            testdata <-rbind(x2,y1)

            tr_va <- rbind(x1,y2_tr_va)       ###########
                (tr)an+(va)lide dataset

            #we take train/validate=2:1 ratio
            number <- sample(nrow(tr_va), size =109, replace = F, )
            train <- tr_va[number,]
            validate <- tr_va[-number,]

            rand<-sample(seq(from=2000,to=30000,by=1),1,replace=F)
            set.seed(rand)

            validate_x1<-validate[rownames(validate)%in%rownames(x1),]
            validate_y2<-validate[rownames(validate)%in%rownames(y2),]

            model <- svm(x=train[,-1],y=train[,1],type =
'C-classification', kernel = 'radial')
            train_pred <- predict(object = model, newdata = train[,-1])



            ######
            val_pred <- predict(object = model, newdata = validate[,-1])

            tab <- table(val_pred, validate[,1])
            accu<-sum(diag(tab))/sum(tab)
            accuracy<-rbind(accuracy,accu)

            false_n_p<-cbind(tab[1,2]/dim(validate_x1)[1],tab[2,1]/dim(v
alidate_y2)[1])

            false_n_p_rates<-rbind(false_n_p_rates,false_n_p)
```

```
                        ###########
        }

                        # test
                        test <- predict(object = model, newdata = testdata[,-1])

                        true_positive<-names(test[test==1])
                        true_negative<-names(test[test==4])

                        positive<-rbind(positive,as.matrix(true_positive))
                        negative<-rbind(negative,as.matrix(true_negative))


                        #how often are group 2 FBX genes discovered at one run
                        true_x2<-length(true_positive[true_positive%in%rownames(x2)]
)

                        true_x2_rate<-true_x2/dim(x2)[1]

                        true_y1<-length(true_positive[true_positive%in%rownames(y1)]
)

                        true_y1_rate<-true_y1/dim(y1)[1]

                        true_prediction_rate<-cbind(true_x2_rate,true_y1_rate)

                        true_prediction<-rbind(true_prediction,true_prediction_rate)


                        }

                    #
                    mean_accuracy<-rbind(mean_accuracy,mean(accuracy))

                    mean_true_prediction<-rbind(mean_true_prediction,colMeans(true_p
rediction))
                    mean_false_n_p_rates<-rbind(mean_false_n_p_rates,colMeans(false_
n_p_rates))


                    constant_positive<-predict_stat(positive)
                    constant_negative<-predict_stat(negative)

                    constant_positive_prediction<-rbind(constant_positive_prediction
,constant_positive)


                    constant_negative_prediction<-rbind(constant_negative_prediction
,constant_negative)

            }
```

```
            svm_m2_negative_predictions<-as.matrix(table(rownames(constant_negative_
prediction)))
            svm_m2_negative_predictions<-svm_m2_negative_predictions[svm_m2_negative
_predictions[,1]>9,]
            svm_negative_names<-names(svm_m2_negative_predictions)

            svm_m2_positive_predictions<-as.matrix(table(rownames(constant_positive_
prediction)))
            svm_m2_positive_predictions<-svm_m2_positive_predictions[svm_m2_positive
_predictions[,1]>9,]
            svm_positive_names<-names(svm_m2_positive_predictions)

            svm_mean_accuracy<-mean_accuracy
            svm_mean_true_prediction<-mean_true_prediction
            svm_mean_false_n_p_rates<-mean_false_n_p_rates



# Machine learning 2: ANN (artificial neural network)

            #read in data
            data0 <-
read.table("Data_S3_fbx_multi_dimensional_features.tab",header= T)
            data0 <-data0[,-1]

            data0<-data0[,-c(6:11,14:23)]
            colnames(data0)

            data0<-data0[order(data0$group),]
            scaleddata<-scale(data0)
            colnames(scaleddata)

            #group data
            x<-scaleddata[1:82,]
            y<-scaleddata[83:692,]

            x_group<-rep(1,dim(x)[1]) #taking Groups 1 and 2 as known FBXes,
labelled as 1
            y_group<-rep(0,dim(y)[1]) #taking Groups 3 and 4 as unknown FBXes,
labelled as 0

            x<-cbind(x_group,x[,-1])
            y<-cbind(y_group,y[,-1])

            colnames(x)<-gsub("x_group","activity",colnames(x))

            colnames(y)<-gsub("y_group","activity",colnames(y))

            x<-data.frame(x)
            y<-data.frame(y)
```

```
x1<-x[1:41,]
x2<-x[42:dim(x)[1],]
y1<-y[1:470,]
y2<-y[471:dim(y)[1],]

#library
library("neuralnet")

#prediction
constant_positive_prediction<-c()
constant_negative_prediction<-c()


mean_accuracy<-c()
mean_true_prediction<-c()
mean_false_n_p_rates<-c()


for(j in 1:10){

        accuracy<-c()
        positive<-c()
        negative<-c()
        true_prediction<-c()
        false_n_p_rates<-c()

        for (i in 1:1000){


            sample <-sample(nrow(y2),size=123,replace=F,)
            y2_tr_va <- y2[sample, ] # validate
            y2_test<-y2[-sample,]

            #we treat groups 2 and 3 FBX genes as unknown for testing
            testdata <-rbind(x2,y1)

            tr_va <- rbind(x1,y2_tr_va)       ###########
                (tr)an+(va)lide dataset

            #we take train/validate=2:1 ratio
            number <- sample(nrow(tr_va), size =109, replace = F, )
            train <- tr_va[number,]
            validate <- tr_va[-number,]


            rand<-sample(seq(from=2000,to=30000,by=1),1,replace=F)
            set.seed(rand)

            validate_x1<-validate[rownames(validate)%in%rownames(x1),]
```

```
                        validate_y2<-validate[rownames(validate)%in%rownames(y2),]

                        nn <- neuralnet(activity~., data=train, hidden=c(10,2), 
linear.output=FALSE, threshold=0.01)

                        val_output <- compute(nn, validate[, -1])
                        val_pred <- val_output$net.result
                        val_pred <- ifelse(val_pred>0.5, 1, 0)
                        tab <- table(val_pred, validate[,1])
                        accu<-1-sum(diag(tab))/sum(tab)
                        accuracy<-rbind(accuracy,accu)

                        false_n_p<-cbind(tab[1,2]/dim(validate_x1)[1],tab[2,1]/dim(v
alidate_y2)[1])

                        false_n_p_rates<-rbind(false_n_p_rates,false_n_p)

                         #### final test ####

                        # test
                        test <- compute(nn, testdata[, -1])
                        test_prediction <- test$net.result
                        test_prediction_adj <- ifelse(test_prediction>0.5, 1, 0)

                        rownames(test_prediction_adj)<-rownames(test_prediction)
                        true_positive<-names(test_prediction_adj[test_prediction_adj
[,1]>0,])

                        true_negative<-names(test_prediction_adj[test_prediction_adj
[,1]<1,])


                        positive<-rbind(positive,as.matrix(true_positive))
                        negative<-rbind(negative,as.matrix(true_negative))


                        #how often are group 2 FBX genes discovered at one run
                        true_x2<-length(true_positive[true_positive%in%rownames(x2)]
)

                        true_x2_rate<-true_x2/dim(x2)[1]

                        true_y1<-length(true_positive[true_positive%in%rownames(y1)]
)

                        true_y1_rate<-true_y1/dim(y1)[1]

                        true_prediction_rate<-cbind(true_x2_rate,true_y1_rate)

                        true_prediction<-rbind(true_prediction,true_prediction_rate)


                        }

                mean_accuracy<-rbind(mean_accuracy,mean(accuracy))
```

```
                mean_true_prediction<-rbind(mean_true_prediction,colMeans(true_predi
ction))
                mean_false_n_p_rates<-rbind(mean_false_n_p_rates,colMeans(false_n_p_
rates))

                constant_positive<-predict_stat(positive)
                constant_negative<-predict_stat(negative)

                constant_positive_prediction<-rbind(constant_positive_prediction,con
stant_positive)

                constant_negative_prediction<-rbind(constant_negative_prediction,con
stant_negative)


            }


            ann_m2_negative_predictions<-as.matrix(table(rownames(constant_negative_
prediction)))
            ann_m2_negative_predictions<-ann_m2_negative_predictions[ann_m2_negative
_predictions[,1]>9,]
            ann_negative_names<-names(ann_m2_negative_predictions)

            ann_m2_positive_predictions<-as.matrix(table(rownames(constant_positive_
prediction)))
            ann_m2_positive_predictions<-ann_m2_positive_predictions[ann_m2_positive
_predictions[,1]>9,]
            ann_positive_names<-names(ann_m2_positive_predictions)

            ann_mean_accuracy<-1-mean_accuracy
            ann_mean_true_prediction<-mean_true_prediction
            ann_mean_false_n_p_rates<-mean_false_n_p_rates



# Machine learning 3: RF (random forest )

        #read in data
        data0 <- read.table("Data_S3_fbx_multi_dimensional_features.tab",header= T)
        data0 <-data0[,-1]

        data0<-data0[,-c(6:11,14:23)]
        colnames(data0)

        #group data
        x1 <- data0[data0$group==1,]
        x2 <- data0[data0$group==2,]
        y1 <- data0[data0$group==3,]
```

```
y2 <- data0[data0$group==4,]

#library
#library(naniar)
library(randomForest)

#prediction
constant_positive_prediction<-c()
constant_negative_prediction<-c()

mean_accuracy<-c()
mean_true_prediction<-c()
mean_false_n_p_rates<-c()



for(j in 1:10){


        accuracy<-c()
        positive<-c()
        negative<-c()
        true_prediction<-c()
        false_n_p_rates<-c()


        for (i in 1:1000){

                sample <-sample(nrow(y2),size=123,replace=F,)
                y2_tr_va <- y2[sample, ] # validate
                y2_test<-y2[-sample,]

                #we treat groups 2 and 3 FBX genes as unknown for testing
                testdata <-rbind(x2,y1)

                tr_va <- rbind(x1,y2_tr_va)       ###########
                    (tr)an+(va)lide dataset

                #we take train/validate=2:1 ratio
                number <- sample(nrow(tr_va), size =109, replace = F, )
                train <- tr_va[number,]
                validate <- tr_va[-number,]

                rand<-sample(seq(from=2000,to=30000,by=1),1,replace=F)
                set.seed(rand)

                validate_x1<-validate[rownames(validate)%in%rownames(x1),]
                validate_y2<-validate[rownames(validate)%in%rownames(y2),]
```

```
###########

train$group <- as.factor(train$group)
#features <- setdiff( x = names(train), y = "group")

#tuneRF(x = train[features], y = train$group, mtryStart =
    1, ntreeTry = 500,)  ### mtry=10 gives us lowest error

#### run model
model <- randomForest(group ~., data=train, ntree=500,
    mtry=4,       ### ntree=500 as default
                  do.trace=100, na.action = na.roughfix)


######
val_pred <- predict(object = model, newdata = validate[,-1])

tab <- table(val_pred, validate[,1])
accu<-sum(diag(tab))/sum(tab)
accuracy<-rbind(accuracy,accu)

false_n_p<-cbind(tab[1,2]/dim(validate_x1)[1],tab[2,1]/dim(v
alidate_y2)[1])

false_n_p_rates<-rbind(false_n_p_rates,false_n_p)


###########


# test

test <- predict(model, newdata = testdata[,-1], type =
"class")



true_positive<-names(test[test==1])
true_negative<-names(test[test==4])

positive<-rbind(positive,as.matrix(true_positive))
negative<-rbind(negative,as.matrix(true_negative))


#how often are group 2 FBX genes discovered at one run
true_x2<-length(true_positive[true_positive%in%rownames(x2)]
)

true_x2_rate<-true_x2/dim(x2)[1]

true_y1<-length(true_positive[true_positive%in%rownames(y1)]
)

true_y1_rate<-true_y1/dim(y1)[1]
```

```
                        true_prediction_rate<-cbind(true_x2_rate,true_y1_rate)

                        true_prediction<-rbind(true_prediction,true_prediction_rate)


                        }

                    #
                    mean_accuracy<-rbind(mean_accuracy,mean(accuracy))

                    mean_true_prediction<-rbind(mean_true_prediction,colMeans(true_p
rediction))
                    mean_false_n_p_rates<-rbind(mean_false_n_p_rates,colMeans(false_
n_p_rates))

                    constant_positive<-predict_stat(positive)
                    constant_negative<-predict_stat(negative)

                    constant_positive_prediction<-rbind(constant_positive_prediction
,constant_positive)

                    constant_negative_prediction<-rbind(constant_negative_prediction
,constant_negative)

            }


            rf_m2_negative_predictions<-as.matrix(table(rownames(constant_negative_p
rediction)))
            rf_m2_negative_predictions<-rf_m2_negative_predictions[rf_m2_negative_pr
edictions[,1]>9,]
            rf_negative_names<-names(rf_m2_negative_predictions)

            rf_m2_positive_predictions<-as.matrix(table(rownames(constant_positive_p
rediction)))
            rf_m2_positive_predictions<-rf_m2_positive_predictions[rf_m2_positive_pr
edictions[,1]>9,]
            rf_positive_names<-names(rf_m2_positive_predictions)

            rf_mean_accuracy<-mean_accuracy
            rf_mean_true_prediction<-mean_true_prediction
            rf_mean_false_n_p_rates<-mean_false_n_p_rates

    ############# Comparison

    #normalize prediction accuracy by the total number of prediciton

    rf_mean_accuracy_nm<-rf_mean_accuracy/(length(rf_negative_names)+length(rf_posit
```

```
ive_names))

    ann_mean_accuracy_nm<-ann_mean_accuracy/(length(ann_negative_names)+length(ann_p
ositive_names))

    svm_mean_accuracy_nm<-svm_mean_accuracy/(length(svm_negative_names)+length(svm_p
ositive_names))


    rf_mean_accuracy_nm_df<-data.frame(Measure=rf_mean_accuracy_nm, Group="RF")
    ann_mean_accuracy_nm_df<-data.frame(Measure=ann_mean_accuracy_nm, Group="ANN")
    svm_mean_accuracy_nm_df<-data.frame(Measure=svm_mean_accuracy_nm, Group="SVM")

    accuracy_nm_df<-rbind(rf_mean_accuracy_nm_df,ann_mean_accuracy_nm_df,svm_mean_ac
curacy_nm_df)

    attach(accuracy_nm_df)

    pdf("Figure5A_accuracy_nm_df.pdf", family="Times",height=7, width=2.5)
        boxplot(accuracy_nm_df$Measure ~ accuracy_nm_df$Group, ylim=c(0,0.005),

        cex.names=0.3,main="clusteri_wgd_ks_comparison",col=c("red","yellow"),outlin
e=FALSE)
    dev.off()


    t.test(ann_mean_accuracy_nm, rf_mean_accuracy_nm, alternative = c("greater"),
mu = 0,
              paired = FALSE, var.equal = FALSE, conf.level = 0.95)

                            #
               #               Welch Two Sample t-test

               #           data:  ann_mean_accuracy_nm and rf_mean_accuracy_nm
               #           t = 1367.7, df = 17.732, p-value < 2.2e-16
               #           alternative hypothesis: true difference in means is
                   greater than 0
               #           95 percent confidence interval:
               #            0.0007548802          Inf
               #           sample estimates:
               #             mean of x   mean of y
               #           0.003166187 0.002410347


    #

    t.test(ann_mean_accuracy_nm, svm_mean_accuracy_nm, alternative = c("greater"),
mu = 0,
              paired = FALSE, var.equal = FALSE, conf.level = 0.95)
```

```
                                   #
               #                   Welch Two Sample t-test

               #           data:  ann_mean_accuracy_nm and svm_mean_accuracy_nm
               #           t = 2283.5, df = 15.321, p-value < 2.2e-16
               #           alternative hypothesis: true difference in means is
                   greater than 0
               #           95 percent confidence interval:
               #            0.001122137         Inf
               #           sample estimates:
               #             mean of x   mean of y
               #           0.003166187 0.002043189


    #####################



    library(VennDiagram)

    ########



    venn.plot <- draw.pairwise.venn(
    area1 = length(ann_negative_names),
    area2 = length(svm_negative_names),
    cross.area =
length(ann_negative_names[ann_negative_names%in%svm_negative_names]),
    category = c("ann", "svm"),
    fill = c("blue", "red"),
    lty = "blank",
    cex = 2,
    cat.cex = 2,
    cat.pos = c(285, 105),
    cat.dist = 0.09,
    cat.just = list(c(-1, -1), c(1, 1)),
    ext.pos = 30,
    ext.dist = -0.05,
    ext.length = 0.85,
    ext.line.lwd = 2,
    ext.line.lty = "dashed"
    );
    grid.draw(venn.plot);
    grid.newpage();

    pdf("Figure5C1_ann_svm_negative_venndiagram.pdf",family="Times",height=10,width=
10)

        grid.draw(venn.plot);
    dev.off();
```

```
    # positive ann vs svm

    venn.plot <- draw.pairwise.venn(
    area1 = length(ann_positive_names),
    area2 = length(svm_positive_names),
    cross.area =
length(ann_positive_names[ann_positive_names%in%svm_positive_names]),
    category = c("ann", "svm"),
    fill = c("blue", "red"),
    lty = "blank",
    cex = 2,
    cat.cex = 2,
    cat.pos = c(285, 105),
    cat.dist = 0.09,
    cat.just = list(c(-1, -1), c(1, 1)),
    ext.pos = 30,
    ext.dist = -0.05,
    ext.length = 0.85,
    ext.line.lwd = 2,
    ext.line.lty = "dashed"
    );
    grid.draw(venn.plot);
    grid.newpage();

    pdf("Figure5B1_ann_svm_positive_venndiagram.pdf",family="Times",height=10,width=
10)

        grid.draw(venn.plot);
    dev.off();


    ###


    venn.plot <- draw.pairwise.venn(
    area1 = length(ann_negative_names),
    area2 = length(rf_negative_names),
    cross.area =
length(ann_negative_names[ann_negative_names%in%rf_negative_names]),
    category = c("ann", "rf"),
    fill = c("blue", "red"),
    lty = "blank",
    cex = 2,
    cat.cex = 2,
    cat.pos = c(285, 105),
    cat.dist = 0.09,
    cat.just = list(c(-1, -1), c(1, 1)),
    ext.pos = 30,
    ext.dist = -0.05,
```

```
    ext.length = 0.85,
    ext.line.lwd = 2,
    ext.line.lty = "dashed"
    );
    grid.draw(venn.plot);
    grid.newpage();

    pdf("Figure5C2_ann_rf_negative_venndiagram.pdf",family="Times",height=10,width=1
0)

        grid.draw(venn.plot);
    dev.off();


    #

    venn.plot <- draw.pairwise.venn(
    area1 = length(ann_positive_names),
    area2 = length(rf_positive_names),
    cross.area =
length(ann_positive_names[ann_positive_names%in%rf_positive_names]),
    category = c("ann", "rf"),
    fill = c("blue", "red"),
    lty = "blank",
    cex = 2,
    cat.cex = 2,
    cat.pos = c(285, 105),
    cat.dist = 0.09,
    cat.just = list(c(-1, -1), c(1, 1)),
    ext.pos = 30,
    ext.dist = -0.05,
    ext.length = 0.85,
    ext.line.lwd = 2,
    ext.line.lty = "dashed"
    );
    grid.draw(venn.plot);
    grid.newpage();

    pdf("Figure5B2_ann_rf_positive_venndiagram.pdf",family="Times",height=10,width=1
0)

        grid.draw(venn.plot);
    dev.off();
```
