# Supplementary material for: A Machine Learning Approach to Prioritizing Functionally Active F-box Members in Arabidopsis thaliana
Source: Front Plant Sci. 2021 May 28;12:639253. doi: 10.3389/fpls.2021.639253 (PMC8192846; doi:10.3389/fpls.2021.639253)
Supplement: Supplementary file 8 [file Presentation_3.PDF]

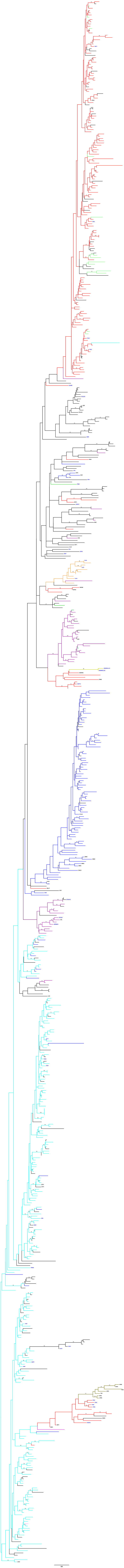
0.5
